# Nurses’ work engagement and perceived public health impact in post-pandemic China: a cross-sectional pathway study of knowledge translation, practice improvement, and community-academic partnerships

**DOI:** 10.3389/fpubh.2026.1926797

**Published:** 2026-09-01

**Authors:** Shan Wang, Chunmei Li, Qinfang Liu

**Affiliations:** 1 Department of Emergency, Ningbo Ninth Hospital, Ningbo, Zhejiang, China; 2 Department of Hand-Foot and Microreconstructive Surgery, Qilu Hospital (Qingdao), Cheeloo College of Medicine, Shandong University, Qingdao, Shandong, China

**Keywords:** community-academic partnerships, knowledge translation, nurses’ work engagement, perceived public health impact, PLS-SEM, post-pandemic nursing, practice improvement

## Abstract

**Background:**

Public health nursing remains important to post-pandemic health-system resilience because nurses connect clinical care with prevention and community support. However, it remains unclear how nurses’ work engagement is associated with perceived public health contribution through evidence use and practice improvement.

**Objective:**

This study examined associations among nurses’ work engagement, community–academic partnerships, knowledge translation, practice improvement, and nurses’ perceived public health impact. Methods: A quantitative cross-sectional survey was conducted among registered nurses in mainland China between 15 January 2026 and 31 March 2026. A total of 450 questionnaires were distributed, and 420 valid responses were included in the analysis. The five constructs were measured using multi-item five-point Likert scales. Data were analyzed using descriptive statistics, reliability and validity assessment, hierarchical regression, partial least squares structural equation modeling, bootstrapped indirect-effect analysis, subgroup analysis, and sensitivity checks.

**Results:**

The five constructs showed acceptable internal consistency, convergent validity, and discriminant validity. Nurses’ work engagement was positively associated with knowledge translation; knowledge translation was positively associated with practice improvement; and practice improvement was positively associated with perceived public health impact. Community–academic partnerships were positively associated with both knowledge translation and practice improvement.

**Conclusion:**

Nurses’ work engagement was indirectly associated with perceived public health impact through knowledge translation and practice improvement. These findings support an integrated, association-based pathway linking workforce engagement, evidence use, academic–practice collaboration, and practice improvement, while the cross-sectional design precludes causal inference.

## 1 Introduction

While prevention, health education, surveillance, community follow-up, and public health response have long been established nursing functions, the COVID-19 pandemic significantly amplified their visibility and accelerated their integration into conventional clinical care. Public health nurses and community-facing nurses were central to vaccination, contact tracing, risk communication, and continuity of care during the pandemic, yet many of these expanded responsibilities have remained relevant in the post-pandemic period (1). As health systems move from emergency response to long-term recovery, nursing practice is increasingly expected to connect clinical care with community health needs, preparedness capacity, and population-oriented service delivery (2). This shift has made nurses’ work engagement a practical concern rather than only a workforce attitude. Empirical literature on occupational health consistently demonstrates that engaged healthcare professionals exhibit heightened levels of energy, persistence, and clinical responsibility (3, 4). Furthermore, this deeply rooted psychological state fosters a willingness to engage in general quality improvement initiatives, encompassing voluntary unit-level problem-solving and institutional committee participation. It remains strictly necessary to distinguish these broad, ad-hoc organizational citizenship behaviors from the specific Practice Improvement construct evaluated in the present study. Within our proposed conceptual framework, Practice Improvement is operationalized as the targeted, measurable enhancement of daily clinical workflows, patient education, and community follow-up directly resulting from the deliberate translation of academic evidence into routine care. Both foundational engagement and structured practice advancements are exceptionally vital when post-pandemic environments require rapid adaptation and evidence-informed decision-making. The Future of Nursing 2020–2030 report emphasized that nurses are positioned to promote health equity, redesign care, and lead system change across clinical and community settings (5). In this context, nurses’ work engagement may serve as an important starting point for public health-oriented practice improvement, especially when nurses are expected to transform professional commitment into sustained changes in daily work.

However, engagement alone may not be sufficient to produce visible practice value. During and after the pandemic, nurses often had to translate new guidelines, infection control procedures, community education messages, and changing public health policies into workable routines. Studies on community health nurses during COVID-19 showed that their roles expanded across prevention, education, coordination, and direct community response, but also revealed challenges related to workload, resource constraints, and rapidly changing practice expectations (6). These findings suggest that the effect of nurses’ work engagement depends partly on whether nurses have the knowledge, support, and organizational conditions needed to convert motivation into evidence-informed action.

Work engagement theory provides a useful basis for understanding this process. Engagement is commonly conceptualized through vigor, dedication, and absorption, reflecting a positive and persistent work-related state rather than a temporary mood (4). In nursing, this distinction matters because engaged nurses may be more willing to seek evidence, participate in improvement work, and adapt to changing practice demands. Recent empirical work has also linked nurses’ work engagement with evidence-based practice implementation, suggesting that engagement can support the movement from professional motivation to evidence use when practice barriers are manageable (7).

Knowledge translation is a second key mechanism in this pathway. Evidence-based nursing requires more than awareness of research findings; it requires nurses to access evidence, interpret it, adapt it to local conditions, and apply it in routine care. Evidence-based practice competencies for nurses emphasize that research evidence, clinical expertise, patient preferences, and practice context must be integrated into decision-making rather than treated as separate domains (8). The Knowledge-to-Action framework similarly conceptualizes knowledge translation as a cycle of adapting knowledge to context, assessing barriers, implementing interventions, monitoring use, and sustaining change (9). For post-pandemic nursing, this framework is particularly relevant because practice knowledge often has to be adjusted to local staffing, patient needs, community resources, and institutional constraints. The translation of knowledge into practice is also shaped by organizational and partnership conditions. Implementation research has long argued that best evidence does not automatically become best practice, because change depends on social, organizational, and system-level mechanisms (10). Community–academic partnerships are one such mechanism. These partnerships can connect frontline nursing teams with academic expertise, research resources, joint training, and practice improvement projects. A systematic review of community–academic partnerships emphasized shared goals, trust, bidirectional exchange, and sustained collaboration as core features of effective partnership work (11). In nursing specifically, academic-practice partnerships have been discussed as a promising route for strengthening evidence-based nursing education and practice, particularly when collaboration is linked to real practice problems rather than symbolic institutional cooperation (12).

Practice improvement represents the proximal outcome of this process. When knowledge translation is effective, nurses may be better able to improve workflow, patient education, interdisciplinary communication, follow-up support, and participation in quality improvement activities. These are not merely internal service indicators; they are also part of how nursing contributes to broader public health-oriented care. At the same time, it is important to distinguish perceived public health impact from objective population-level outcomes. Implementation outcome frameworks caution that implementation outcomes, service outcomes, and population health outcomes are related but conceptually distinct (13). Therefore, in this study, perceived public health impact refers to nurses’ perceived contribution to health awareness, preventive care, vulnerable population support, continuity of care, and responsiveness to public health needs, rather than direct epidemiological change. Despite extensive research on nurses’ work engagement, evidence-based practice, knowledge translation, and academic-practice collaboration as isolated phenomena, their theoretical integration remains fragmented. Specifically, the conceptual mechanism through which nurses’ psychological engagement translates into tangible, public health-oriented practice value remains underdeveloped (14). This study addresses this critical gap by theoretically bridging Work Engagement Theory with the Knowledge-to-Action framework. By proposing a unified conceptual model, this research foregrounds the theoretical contribution of positioning knowledge translation and practice improvement as sequential mediators. Accordingly, the present study tested a quantitative pathway model in which Nurses’ work engagement was associated with Knowledge Translation, Knowledge Translation was associated with Practice Improvement, and Practice Improvement was associated with Perceived Public Health Impact. Community–Academic Partnerships were modeled as an exogenous organizational partnership construct associated with Knowledge Translation and Practice Improvement.

Accordingly, this study aimed to examine the associations among Nurses’ work engagement, Community–Academic Partnerships, Knowledge Translation, Practice Improvement, and Perceived Public Health Impact among registered nurses in the post-pandemic era. The study further assessed whether Knowledge Translation and Practice Improvement formed an indirect pathway linking Nurses’ work engagement with Perceived Public Health Impact, and whether Community–Academic Partnerships contributed to this pathway by supporting Knowledge Translation and Practice Improvement.

## 2 Materials and methods

### 2.1 Study design

**Figure 1:**
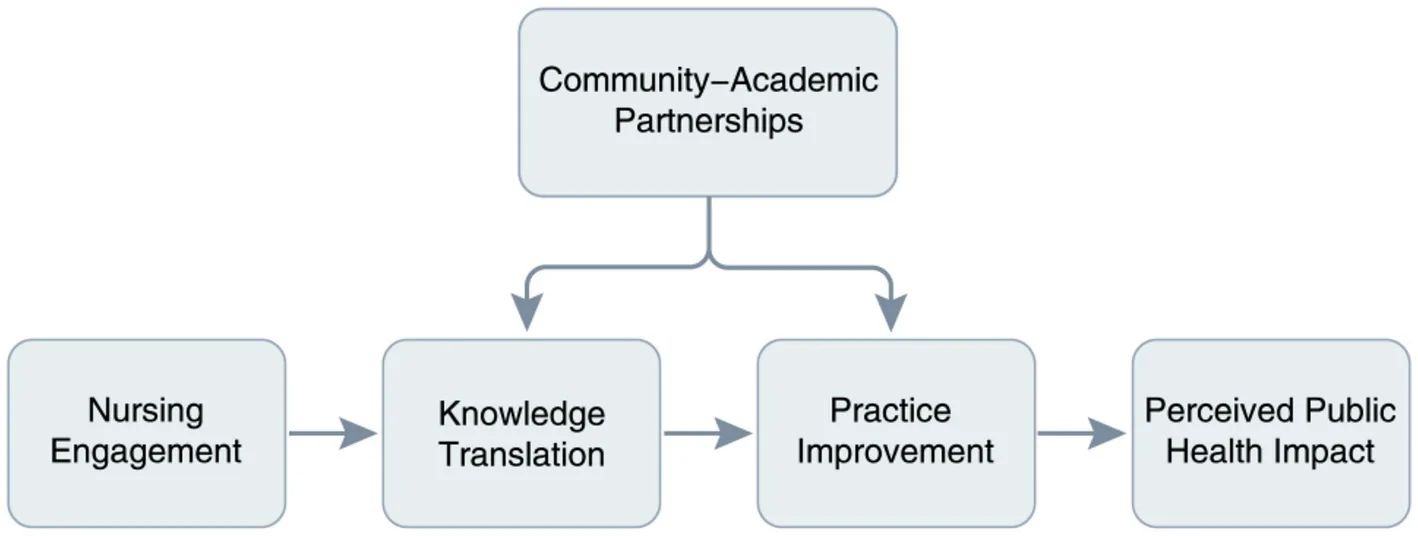
Conceptual and statistical framework of the study.

This study used a quantitative cross-sectional survey design to examine how nurses’ work engagement, community–academic partnerships, knowledge translation, and practice improvement were associated with nurses’ perceived public health impact in the post-pandemic era. A cross-sectional design was appropriate because the study assessed naturally occurring differences in nurses’ work engagement, partnership exposure, knowledge translation behavior, and practice improvement at a defined point in time rather than evaluating an intervention. The reporting structure followed the STROBE checklist for observational cross-sectional studies (15). The conceptual and statistical framework is presented in Figure 1.

The pre-specified model positioned Nurses’ work engagement as the initial individual-level predictor, Knowledge Translation as the first pathway construct, Practice Improvement as the proximal practice outcome, and Perceived Public Health Impact as the final outcome. Community–Academic Partnerships were modeled as an exogenous organizational partnership construct with direct paths to Knowledge Translation and Practice Improvement.

### 2.2 Study setting and participants

The study included registered nurses working in public hospitals, community health centers, public health nursing units, and academic–practice collaborative nursing settings across 12 healthcare institutions, primarily in the eastern coastal provinces of mainland China. Data were collected from 15 January 2026 to 31 March 2026 through an electronic questionnaire platform; recruitment and consent began after ethical approval on 10 January 2026. A purposive convenience sampling strategy was used because the study required nurses with relevant clinical, community-oriented, knowledge-translation, or academic–practice collaboration experience. Nursing department coordinators, professional nursing groups, and collaboration networks distributed the survey link. Coordinators did not determine eligibility or access individual responses, and no incentives were offered.

Participants were eligible if they were registered nurses aged 18 years or older, currently employed in a clinical or public health-related nursing role, had at least 6 months of continuous nursing work experience after the COVID-19 public health emergency phase, and provided electronic informed consent. Nursing interns, nursing students without independent clinical responsibility, retired nurses, nurse’s not currently engaged in clinical or public health-related nursing work, and respondents with invalid questionnaire submissions were excluded.

**Figure 2:**
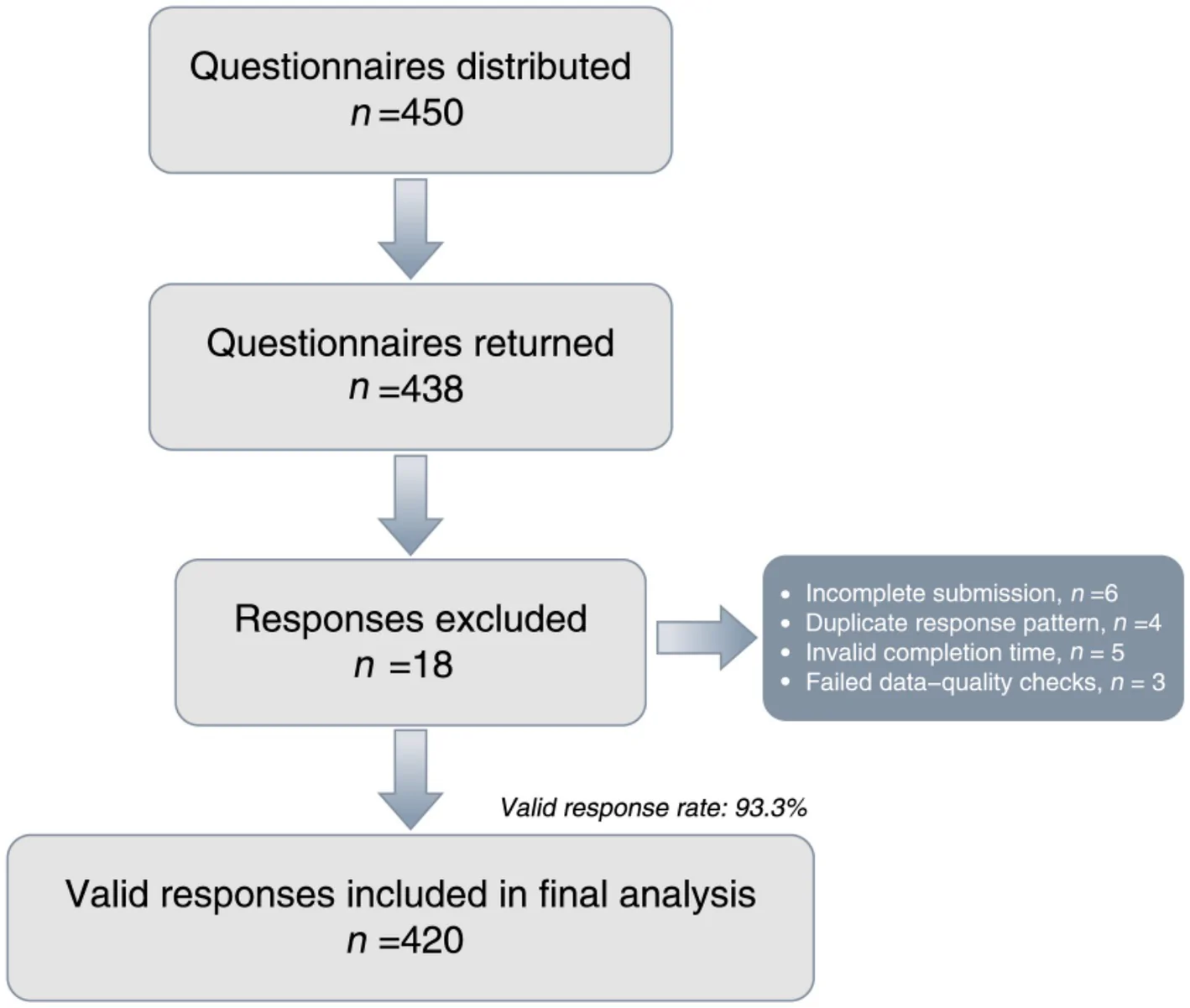
Participant screening and analytic sample flow.

A total of 450 questionnaires were distributed, and 438 questionnaires were returned. After data screening, 18 responses were excluded because of incomplete submission, duplicate response patterns, invalid completion time, or failed data-quality checks. The final analytic sample included 420 valid responses, corresponding to a valid response rate of 93.3% based on all distributed questionnaires. The high valid response rate may reflect the efficiency of distributing the survey through established nursing and academic-practice networks. Although participation remained entirely voluntary, anonymous, and structurally decoupled from formal workplace evaluations, localized administrative encouragement to participate may have subtly contributed to this elevated return rate. The participant screening and analytic sample flow is shown in Figure 2.

A total of 450 questionnaires were distributed and 438 were returned. Eighteen responses were excluded because of incomplete submission, duplicate response patterns, invalid completion time, or failed data-quality checks. The final analytic sample included 420 valid responses, corresponding to a valid response rate of 93.3% based on all distributed questionnaires.

### 2.3 Sample size determination

Sample size planning considered the requirements of multivariable regression and partial least squares structural equation modeling. The model contained five latent constructs, with the largest number of structural paths directed at one endogenous construct being two. Although the ten-time's rule provided a basic lower-bound reference, the final sample target was set above 400 to support stable estimation of construct loadings, adjusted regression paths, and bootstrapped indirect effects in a mediation-based PLS-SEM model (16).

### 2.4 Measures

The questionnaire contained demographic and professional information followed by five construct domains: Nurses’ work engagement, Community–Academic Partnerships, Knowledge Translation, Practice Improvement, and Perceived Public Health Impact. All construct items used a five-point Likert scale ranging from 1 = strongly disagree to 5 = strongly agree. Higher scores indicated stronger endorsement of the measured construct. Nurses’ work engagement was measured using six items conceptually adapted from the established occupational psychology literature to capture a domain-specific motivational state. Rather than merely reflecting generalized organizational citizenship behavior or passive professional commitment, this construct is deliberately operationalized as an active, persistent psychological investment directed toward clinical responsibilities and evidence-informed practice evolution. While classic frameworks traditionally separate engagement into distinct dimensions of vigor, dedication, and absorption (17), the intense reality of post-pandemic nursing frequently blurs the theoretical boundaries between deep cognitive absorption and proactive dedication to care quality. Consequently, these elements were synthesized into a single latent variable. The items comprehensively assess energy at work, dedication to tasks, persistence under pressure, proactive responsibility for care quality, willingness to integrate improvement activities, and readiness to contribute beyond strictly mandated routines. This unidimensional approach explicitly focuses on the actionable drive necessary for effective knowledge translation.

Community–Academic Partnerships were measured using five items assessing nurses’ exposure to and perceived usefulness of collaboration between healthcare institutions and academic organizations. The items covered joint training, shared practice improvement projects, research-practice communication, access to academic resources, and application of partnership activities in nursing work. The construct was treated as an exogenous organizational partnership factor rather than a moderator. Its measurement was grounded in community–academic partnership literature emphasizing shared goals, bidirectional collaboration, and practice-oriented knowledge exchange (18). Knowledge Translation was measured using six items assessing nurses’ use of research evidence in daily practice. The items covered evidence access, evidence discussion with colleagues, adaptation of evidence to local practice, participation in evidence-based training, evidence-informed decision-making, and translation of new knowledge into routine care. The construct followed the Knowledge-to-Action logic, which describes knowledge translation as a process of adapting, implementing, monitoring, and sustaining evidence use in practice (19).

Practice Improvement was measured using five items reflecting perceived changes in workflow, patient education, interdisciplinary communication, community follow-up, and quality-improvement participation. Perceived Public Health Impact was measured using five items assessing nurses’ perceived contribution to health awareness, preventive care, support for vulnerable populations, continuity of care, and responsiveness to public health needs. Because no single validated instrument fully matched the post-pandemic nursing partnership context, these two outcome-oriented domains were developed *de novo* from evidence-based practice, nursing quality-improvement, and public health nursing domains, then subjected to expert review and pilot testing before the full survey.

The measurement structure and scoring rules are summarized in Table 1. The full questionnaire items and coding rules are provided in Supplementary Table S1. For descriptive interpretation only, mean scores below 3.00 were classified as low, scores from 3.00 to 3.99 as moderate, and scores of 4.00 or above as high. These categories were not treated as validated diagnostic cutoffs and were not used to dichotomize variables in regression or structural equation modeling.

**Table 1:** Measurement structure and scoring rules for the study constructs.

Construct	Code	Number of items	Measurement domains	Response scale	Score calculation	Score interpretation
Nurses’ work engagement	NE	6	Energy at work; dedication to nursing tasks; persistence under pressure; responsibility for care quality; willingness to participate in improvement activities; contribution beyond routine duties	1 = strongly disagree to 5 = strongly agree	Mean score of NE1–NE6	Higher scores indicate stronger nurses’ work engagement
Community–academic partnerships	CAP	5	Joint training; shared practice improvement projects; research-practice communication; access to academic resources; perceived usefulness of partnership activities	1 = strongly disagree to 5 = strongly agree	Mean score of CAP1–CAP5	Higher scores indicate stronger perceived partnership exposure and usefulness
Knowledge translation	KT	6	Evidence access; evidence discussion with colleagues; local adaptation of evidence; evidence application from training, guidelines, or professional resources; evidence-informed decision-making; translation of new knowledge into routine care	1 = strongly disagree to 5 = strongly agree	Mean score of KT1–KT6	Higher scores indicate stronger knowledge translation behavior
Practice improvement	PI	5	Workflow improvement; patient education improvement; interdisciplinary communication; community follow-up; quality improvement participation	1 = strongly disagree to 5 = strongly agree	Mean score of PI1–PI5	Higher scores indicate stronger perceived practice improvement
Perceived public health impact	PHI	5	Health awareness; preventive care; vulnerable population support; continuity of care; responsiveness to public health needs	1 = strongly disagree to 5 = strongly agree	Mean score of PHI1–PHI5	Higher scores indicate stronger perceived public health contribution

All construct scores were calculated as the arithmetic mean of the corresponding item scores. PHI refers to nurses’ perceived public health contribution rather than population-level epidemiological outcomes. For descriptive readability only, mean scores below 3.00 were classified as low, scores from 3.00 to 3.99 as moderate, and scores of 4.00 or above as high. These categories were not treated as validated diagnostic cutoffs and were not used to dichotomize variables in regression or structural equation modeling.

### 2.5 Covariates

The following covariates were collected: age, gender, highest educational level, years of nursing experience, professional title, department type, institution type, hospital level, previous knowledge translation training, and previous involvement in community–academic partnership activities. Years of nursing experience were recorded as a continuous variable and grouped descriptively as <5 years, 5–10 years, 11–20 years, and >20 years. Previous knowledge translation training and previous partnership involvement were coded as binary variables.

### 2.6 Questionnaire development and validity control

The questionnaire was developed in three stages. Because the target population consisted of healthcare professionals in mainland China, the instrument was developed and administered in Mandarin Chinese. First, construct items were drafted from the conceptual model and relevant literature. Second, three nursing management scholars and two senior clinical nurses independently evaluated the 35 initial items on a four-point relevance scale (1 = not relevant, 2 = major revision required, 3 = relevant with minor revision, and 4 = highly relevant). Ratings of 3 or 4 were coded as relevant. I-CVI was calculated as the number of relevant ratings divided by five, so possible values occurred in increments of 0.20. The criterion of I-CVI ≥ 0.78 was operationalized as at least four of five experts agreeing (I-CVI = 0.80) (20, 21). CAP3 and PI3 each received four relevant ratings (I-CVI = 0.80), while the remaining 33 initial items received five relevant ratings (I-CVI = 1.00). Accordingly, S-CVI/UA was 33/35 = 0.943 and S-CVI/Ave was 0.989. The two communication-related items were revised, and eight conceptually redundant items were removed, yielding 27 retained items. The complete five-expert rating matrix is provided in Supplementary Table S5.

Third, a pilot test was conducted with 30 registered nurses who met the eligibility criteria but were excluded from the final analytic sample. The pilot assessed item clarity, response burden, and preliminary item performance. The average completion time was 7.00 ± 0.66 min, with a median of 7.0 min [IQR 6.5–7.5] and a range of 6–8 min. Cronbach’s *α* values were 0.86 for Nurses’ Work Engagement, 0.84 for Community–Academic Partnerships, 0.85 for Knowledge Translation, 0.81 for Practice Improvement, and 0.89 for Perceived Public Health Impact. Corresponding approximate 95% Feldt confidence intervals were 0.765–0.925, 0.727–0.915, 0.748–0.920, 0.676–0.899, and 0.813–0.942, respectively (22). Construct-specific corrected item–total correlation ranges were 0.637–0.669, 0.613–0.658, 0.617–0.661, 0.574–0.612, and 0.709–0.751, respectively. Supplementary Tables S4, S6 report the construct-level and item-level pilot results.

### 2.7 Data collection procedure

To procedurally mitigate potential common method bias, the electronic questionnaire began with an informed consent page explaining the study purpose, voluntary participation, anonymity, estimated completion time, data use, and withdrawal rights. Participants were explicitly assured that there were no predetermined correct answers. This procedural safeguard helps reduce social desirability bias and participant evaluation apprehension. Only participants who selected “I agree to participate” could access the questionnaire. Each device or account was allowed to submit the questionnaire once. The system required all core construct items to be completed. No names, identification numbers, phone numbers, exact residential addresses, or patient-level data were collected. Internet protocol information was used only by the survey platform for duplicate-response restriction and was not exported for analysis. After data collection closed, the dataset was downloaded into password-protected files and evaluated using the pre-specified screening rules.

### 2.8 Data quality control

Invalid responses were excluded according to predefined rules. A completion time below 180 s was used as a conservative exclusion threshold. This threshold was shorter than the approximately 6–8-min pilot summary so that completion time alone would not exclude slower but potentially valid responses; submissions near the lower boundary were evaluated together with duplicate response patterns, straight-lining, and logic checks. Responses were also excluded if consent was absent, construct data were incomplete, or duplicate submission was evident. After screening, construct scores were calculated as the mean of their corresponding item scores.

### 2.9 Missing data handling

Missingness was assessed for demographic variables and all construct items after data export. Respondents with missing values in any primary construct score were excluded from the analytic sample. For covariates, missingness below 5% was handled using complete-case analysis in adjusted regression models. If covariate missingness exceeded 5%, missingness patterns were examined, and missing-indicator sensitivity analysis was planned. No outcome imputation was performed.

### 2.10 Statistical analysis

Statistical analysis was conducted using IBM SPSS Statistics version 29.0 and SmartPLS version 4.1. SPSS was used for data cleaning, descriptive statistics, reliability screening, correlation analysis, and adjusted regression. SmartPLS was used for measurement model assessment, structural path estimation, collinearity diagnosis, discriminant validity testing, and bootstrapped indirect-effect testing. Continuous variables were summarized as mean and standard deviation when approximately normally distributed and as median and interquartile range when markedly skewed. Categorical variables were summarized as frequency and percentage. Normality was assessed using histograms, Q-Q plots, skewness, and kurtosis. Skewness and kurtosis values within approximately ±2.00 were considered acceptable for descriptive parametric analysis. Group comparisons were performed using independent-samples t tests, one-way analysis of variance, Mann–Whitney U tests, Kruskal–Wallis tests, or chi-square tests, as appropriate.

Internal consistency was assessed using Cronbach’s alpha and composite reliability, with values of 0.70 or higher considered acceptable. Convergent validity was assessed using standardized indicator loadings and average variance extracted (AVE). Loadings of 0.70 or higher were preferred, while loadings of 0.60–0.69 were retained only when construct reliability and theoretical relevance were adequate; AVE values of 0.50 or higher were considered acceptable. Discriminant validity was assessed using the Heterotrait–Monotrait ratio, with values below 0.85 indicating adequate discriminant validity (23). Pearson correlations described bivariate associations among the five constructs. Variance inflation factors (VIFs) were examined for structural collinearity and as a limited diagnostic for possible single-source method variance. VIFs below 3.30 were considered reassuring, but they were not interpreted as ruling out common method bias.

Hierarchical multiple regression models were first used to provide covariate-adjusted supplementary estimates. PLS-SEM was then used to estimate the pre-specified latent-variable pathway model while accounting for measurement error and multiple indirect paths. This prediction-oriented approach was selected because the model included five latent constructs and sequential indirect associations (24). The structural model included five direct paths: Nurses’ Work Engagement → Knowledge Translation; Community–Academic Partnerships → Knowledge Translation; Community–Academic Partnerships → Practice Improvement; Knowledge Translation → Practice Improvement; and Practice Improvement → Perceived Public Health Impact. The primary indirect pathway was Nurses’ Work Engagement → Knowledge Translation → Practice Improvement → Perceived Public Health Impact. Secondary indirect pathways were Community–Academic Partnerships → Knowledge Translation → Practice Improvement and Community–Academic Partnerships → Knowledge Translation → Practice Improvement → Perceived Public Health Impact.

Indirect effects were tested using nonparametric bootstrapping with 5,000 resamples. An indirect effect was considered statistically significant when the 95% bootstrap confidence interval did not include zero. Model interpretation used standardized path coefficients, *p* values, bootstrap confidence intervals, and coefficient of determination. The prespecified statistical analysis plan is summarized in Table 2.

**Table 2:** Pre-specified statistical analysis plan.

Analysis step	Purpose	Variables involved	Statistical method	Pres-pecified threshold/decision rule	Software
Descriptive analysis	Describe demographic characteristics and construct scores	Age, gender, education, years of experience, professional title, department type, institution type, hospital level, KT training, CAP involvement, NE, CAP, KT, PI, PHI	Mean and SD; median and IQR; frequency and percentage	Continuous variables reported according to distribution; categorical variables reported as n (%)	SPSS 29.0
Normality assessment	Determine appropriate descriptive and group-comparison methods	Continuous demographic variables and construct scores	Histogram, Q-Q plot, skewness, kurtosis	Skewness and kurtosis within approximately ±2.00 considered acceptable for descriptive parametric analysis	SPSS 29.0
Exploratory group comparison	Explore descriptive differences across demographic and professional groups	NE, CAP, KT, PI, PHI by demographic and professional categories	Independent-samples t test; one-way ANOVA; Mann–Whitney U test; Kruskal–Wallis test; chi-square test	*p* < 0.05 considered statistically significant for exploratory comparison	SPSS 29.0
Internal consistency	Assess reliability of each construct	NE, CAP, KT, PI, PHI	Cronbach’s alpha; composite reliability	≥0.70 considered acceptable	SPSS 29.0; SmartPLS 4.1
Convergent validity	Assess whether items adequately represent their constructs	All latent construct items	Standardized factor loading; average variance extracted	Loading preferably ≥0.70; loading 0.60–0.69 retained only if theoretically justified; AVE ≥ 0.50	SmartPLS 4.1
Discriminant validity	Assess separation between latent constructs	NE, CAP, KT, PI, PHI	Heterotrait–Monotrait ratio	HTMT <0.85 considered acceptable	SmartPLS 4.1
Collinearity diagnosis	Examine predictor overlap before structural interpretation	Predictors in regression and PLS-SEM paths	Variance inflation factor	VIF < 3.30 considered acceptable	SPSS 29.0; SmartPLS 4.1
Correlation analysis	Examine preliminary bivariate associations among main constructs	NE, CAP, KT, PI, PHI	Pearson correlation analysis	p < 0.05 considered statistically significant	SPSS 29.0
Adjusted regression	Provide covariate-adjusted supplementary estimates	Model 1: KT regressed on NE and CAP; Model 2: PI regressed on KT and CAP; Model 3: PHI regressed on PI; demographic and professional covariates added to adjusted models	Hierarchical multiple regression	p < 0.05 considered statistically significant	SPSS 29.0
Structural model testing	Test the prespecified latent-variable pathway model	NE → KT; CAP → KT; CAP → PI; KT → PI; PI → PHI	Partial least squares structural equation modeling	Path p < 0.05; 95% CI excludes 0; R^2^ reported	SmartPLS 4.1
Mediation analysis	Test primary and secondary indirect pathways	Primary: NE → KT → PI → PHI; secondary: CAP → KT → PI and CAP → KT → PI → PHI	Nonparametric bootstrapping with 5,000 resamples	Indirect effect significant when 95% bootstrap CI excludes zero	SmartPLS 4.1
Sensitivity analysis	Assess robustness of main findings	Main regression and PLS-SEM models	Reanalysis after excluding lowest 5% completion times; subgroup analysis among nurses with KT training; additional adjustment for hospital level, department type, and years of experience	Consistency of direction, magnitude, and statistical significance of main paths assessed	SPSS 29.0; SmartPLS 4.1

NE, Nurses’ work engagement; CAP, Community–academic partnerships; KT, knowledge translation; PI, practice improvement; PHI, perceived public health impact; SD, standard deviation; IQR, interquartile range; AVE, average variance extracted; HTMT, heterotrait–monotrait ratio; VIF, variance inflation factor; PLS-SEM, partial least squares structural equation modeling.

### 2.11 Sensitivity analysis

Three sensitivity analyses were pre-specified. First, the main regression and PLS-SEM models were repeated after excluding respondents with completion times in the lowest 5% of the distribution. Second, the models were repeated among nurses with previous knowledge translation training. Third, the models were repeated after adjustment for hospital level, department type, and years of nursing experience to examine whether the structural paths remained stable across professional background differences.

### 2.12 Ethical considerations

This study was reviewed and approved by the Ethics Committee of Ningbo No. 9 Hospital (Approval No. 2026LIW0009; date of review: 10 January 2026). Electronic informed consent was obtained from all participants before questionnaire completion. Participation was voluntary and anonymous, and respondents were informed that they could refuse to answer questions or withdraw from the survey at any point before completing it without penalty. The questionnaire collected anonymous survey data only and did not involve clinical intervention, biological samples, patient records, or patient-level outcomes. No names, identification numbers, contact details, exact residential addresses, or other directly identifiable personal information were collected. Therefore, the risk to participants was minimal. All procedures were conducted in accordance with the ethical principles of the Declaration of Helsinki and relevant institutional requirements.

### 2.13 Data management and reproducibility

The de-identified dataset, variable codebook, questionnaire, screening log, and statistical syntax are available from the corresponding author upon reasonable request. The cleaned dataset contains 420 rows and includes demographic variables, item-level responses, and computed construct scores. Derived variables were generated according to prespecified scoring rules, and all exclusion decisions, missing-data checks, and score calculations were recorded in the screening log. No personally identifiable information is included in the shared files. The description of nursing and institutional participation followed partnership-reporting principles where relevant (25).

## 3 Results

### 3.1 Participant flow and sample characteristics

A total of 450 questionnaires were distributed between 15 January 2026 and 31 March 2026, and 438 questionnaires were returned. After data screening, 18 responses were excluded because of incomplete submission, duplicate response patterns, invalid completion time, or failed data-quality checks. The final analytic sample included 420 valid responses, corresponding to a valid response rate of 93.3% based on all distributed questionnaires. The participant screening process is shown in Figure 2.

Most participants were female (324/420, 77.1%). The largest age group was 30–39 years (171/420, 40.7%), followed by 20–29 years (111/420, 26.4%), 40–49 years (101/420, 24.0%), and ≥50 years (37/420, 8.8%). More than half held a bachelor’s degree (230/420, 54.8%). By institutional level, 182 nurses (43.3%) worked in tertiary hospitals, 127 (30.2%) in secondary hospitals, and 111 (26.4%) in primary or community-level institutions. Institution-specific counts ranged from 25 to 48 (median 32), and the largest institution contributed 48 of 420 participants (11.4%). Supplementary Table S7 presents the complete distribution. Previous knowledge-translation training was reported by 261 participants (62.1%), and previous involvement in community–academic partnership activities by 225 (53.6%). Table 3 summarizes the sample characteristics.

**Table 3:** Participant characteristics of the analytic sample.

Characteristic	Category	*n*	%
Total sample	—	420	100.0
Gender	Male	96	22.9
Gender	Female	324	77.1
Age group	20–29 years	111	26.4
Age group	30–39 years	171	40.7
Age group	40–49 years	101	24.0
Age group	≥50 years	37	8.8
Highest educational level	Diploma-level qualification	137	32.6
Highest educational level	Bachelor’s degree	230	54.8
Highest educational level	Master’s degree or above	53	12.6
Professional role	Staff nurse	254	60.5
Professional role	Senior staff nurse	114	27.1
Professional role	Nurse manager or educator	52	12.4
Practice setting	Community/public health setting	116	27.6
Practice setting	Medical or surgical department	100	23.8
Practice setting	Outpatient or primary care setting	82	19.5
Practice setting	Emergency or critical care setting	74	17.6
Practice setting	Maternal or child health setting	48	11.4
Institution level	Tertiary hospital	182	43.3
Institution level	Secondary hospital	127	30.2
Institution level	Primary/community-level institution	111	26.4
Previous knowledge translation training	Yes	261	62.1
Previous knowledge translation training	No	159	37.9
Previous community–academic partnership involvement	Yes	225	53.6
Previous community–academic partnership involvement	No	195	46.4

Percentages were calculated using the final analytic sample of 420 valid responses as the denominator. Percentages may not sum to 100.0 because of rounding.

### 3.2 Descriptive results for the study constructs

**Figure 3:**
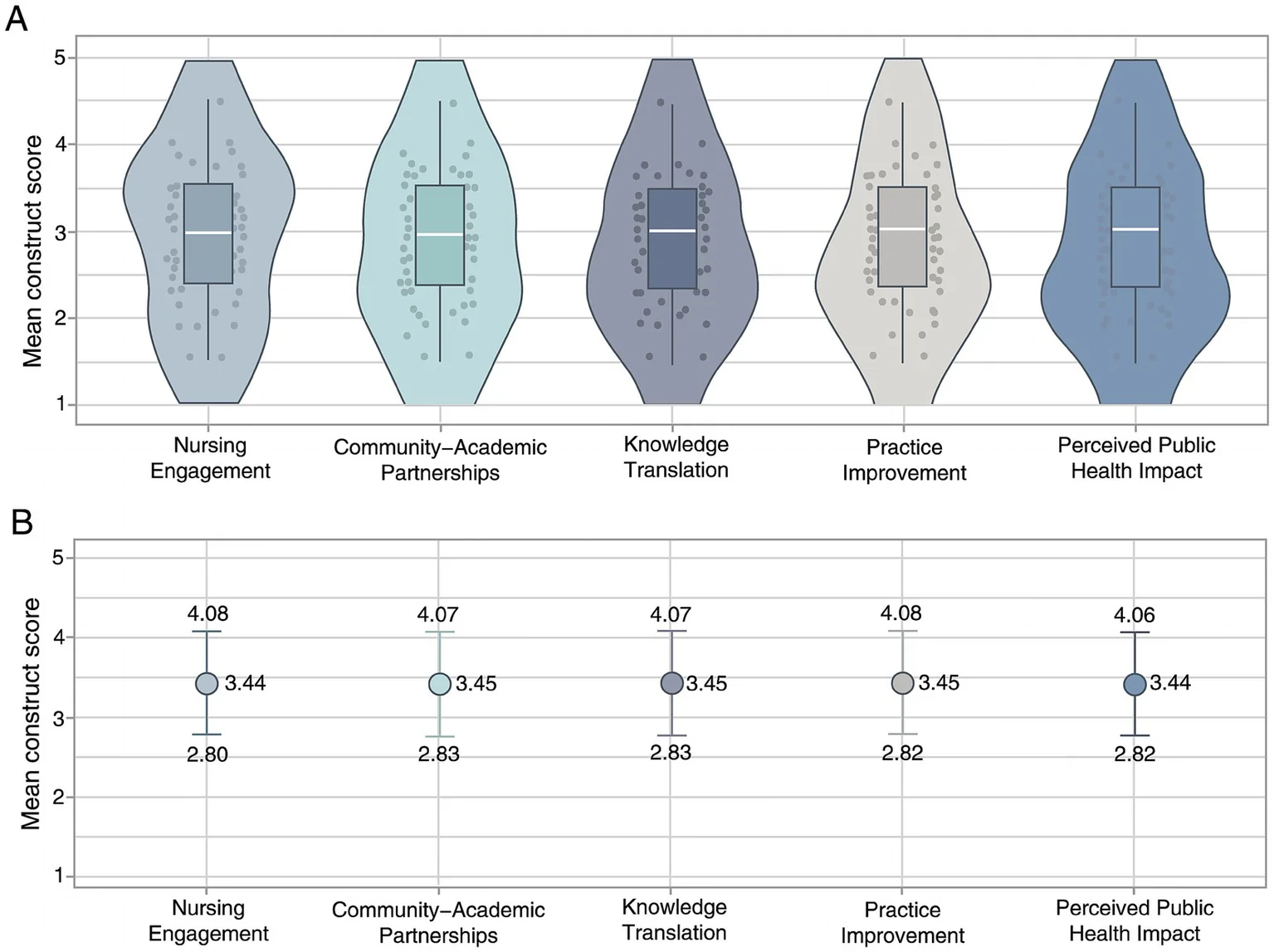
Distribution and descriptive pattern of construct scores. **(A)** Distribution of mean construct scores for Nurses’ Work Engagement, Community–Academic Partnerships, Knowledge Translation, Practice Improvement, and Perceived Public Health Impact. **(B)** Mean construct scores with standard deviations. All constructs were measured on a five-point Likert scale, with higher scores indicating stronger endorsement of the corresponding construct.

The mean scores of the five main constructs were in the moderate range. Nurses’ work engagement had a mean score of 3.44 ± 0.64, Community–Academic Partnerships had a mean score of 3.45 ± 0.62, knowledge Translation had a mean score of 3.45 ± 0.62, Practice Improvement had a mean score of 3.45 ± 0.63, and Perceived Public Health Impact had a mean score of 3.44 ± 0.62. The distribution and descriptive pattern of the five construct scores are shown in Figure 3. Panel A displays the score distributions across constructs, while Panel B presents the mean scores with standard deviations.

Using the pre-specified descriptive categories, most participants scored in the moderate range across all constructs. Moderate scores were observed for 55.2% of participants in Nurses’ work engagement, 57.6% in Community–Academic Partnerships, 60.2% in Knowledge Translation, 55.0% in Practice Improvement, and 57.1% in Perceived Public Health Impact. High scores were observed for approximately one-fifth to one-quarter of participants across the constructs, ranging from 21.9% for Knowledge Translation to 24.5% for Community–Academic Partnerships. These categories were used only for descriptive interpretation and were not used for model estimation.

The distributions of the five construct scores were suitable for the planned analyses. Skewness values ranged from −0.09 to 0.10, and kurtosis values ranged from −0.32 to −0.11, remaining within the pre-specified acceptable range for descriptive parametric analysis. Descriptive statistics and measurement model results are presented in Table 4.

**Table 4:** Construct descriptive statistics and measurement model results.

Construct	Mean ± SD	Med.	IQR	Skew.	Kurt.	Cronbach’s *α*	CR	AVE	Loading range
Nurses’ work engagement	3.44 ± 0.64	3.45	3.00–3.88	−0.04	−0.18	0.892	0.914	0.638	0.781–0.823
Community–academic partnerships	3.45 ± 0.62	3.40	3.00–3.80	0.02	−0.24	0.848	0.894	0.629	0.752–0.813
Knowledge translation	3.45 ± 0.62	3.50	3.00–3.83	−0.09	−0.11	0.873	0.904	0.610	0.759–0.817
Practice improvement	3.45 ± 0.63	3.40	3.00–3.80	0.10	−0.32	0.853	0.898	0.638	0.764–0.821
Perceived public health impact	3.44 ± 0.62	3.40	3.00–3.80	−0.02	−0.27	0.829	0.885	0.607	0.752–0.801

**Figure 4:**
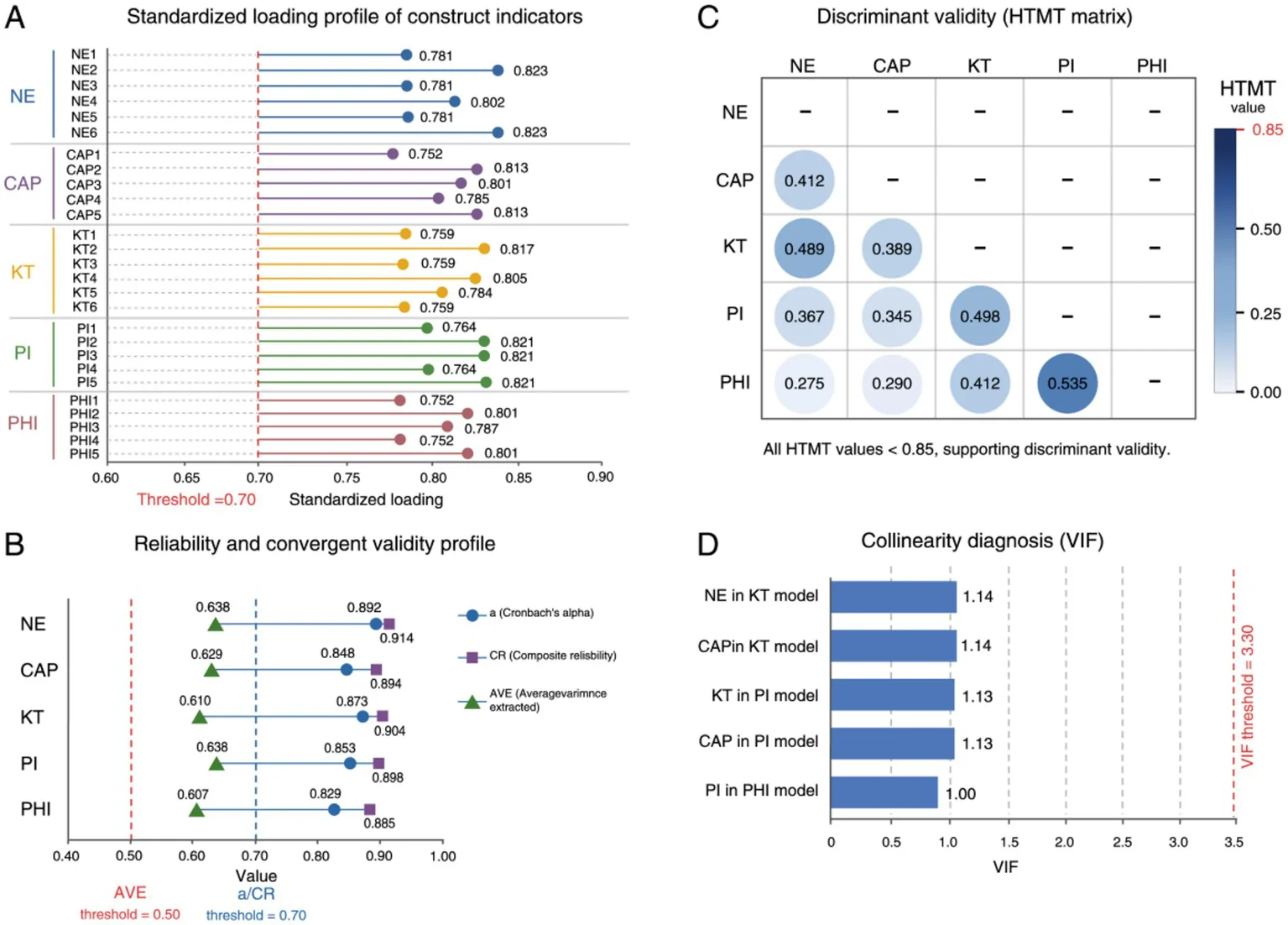
Measurement model quality across latent constructs. **(A)** Standardized loading profile of construct indicators. **(B)** Reliability and convergent validity profile. **(C)** Discriminant validity assessed using the HTMT matrix. **(D)** Collinearity diagnosis based on variance inflation factors.

SD, standard deviation; IQR, interquartile range; CR, composite reliability; AVE, average variance extracted. All construct scores were calculated as the mean of their corresponding items on a five-point Likert scale. Higher scores indicated stronger endorsement of the construct. Cronbach’s α and CR values ≥0.70 indicated acceptable internal consistency. AVE values ≥0.50 indicated acceptable convergent validity. To ensure consistency with Figure 4A and Supplementary Table S2, CR was recalculated as (Σ*λ*)^2^/[(Σ*λ*)^2^ + Σ(1 − *λ*^2^)] and AVE as Σ*λ*^2^/k from the displayed three-decimal standardized loadings.

Figure 4 provides an integrated visual summary of the measurement model quality. As shown in Figure 4A, all standardized indicator loadings exceeded the recommended threshold of 0.70. Figure 4B displays Cronbach’s alpha together with CR and AVE values recalculated from the loadings shown in Figure 4A; all remained above the pre-specified thresholds. The HTMT matrix in Figure 4C showed that all inter-construct HTMT values were below 0.85, indicating acceptable discriminant validity. In addition, the VIF results in Figure 4D ranged from 1.00 to 1.14, below the threshold of 3.30, suggesting that collinearity was not a concern in the structural model.

### 3.3 Measurement model assessment

The measurement model showed acceptable internal consistency, convergent validity, and discriminant validity. Cronbach’s alpha values were 0.892 for Nurses’ Work Engagement, 0.848 for Community–Academic Partnerships, 0.873 for Knowledge Translation, 0.853 for Practice Improvement, and 0.829 for Perceived Public Health Impact. Using the standardized indicator loadings displayed in Figure 4A and Supplementary Table S2, recalculated composite reliability values ranged from 0.885 to 0.914 and AVE values ranged from 0.607 to 0.638. Standardized indicator loadings ranged from 0.752 to 0.823. All reliability and AVE values exceeded the pre-specified thresholds. Because the calculations use the three-decimal loadings available in the submitted figure, minor differences from estimates based on unrounded software output may occur.

Discriminant validity was supported by Heterotrait–Monotrait ratio values below the pre-specified threshold of 0.85. The highest HTMT value was 0.535, observed between Practice Improvement and Perceived Public Health Impact. The remaining HTMT values ranged from 0.275 to 0.530, indicating that the five constructs were empirically distinguishable. The full measurement model results are summarized in Table 4.

### 3.4 Correlations among the main constructs

**Figure 5:**
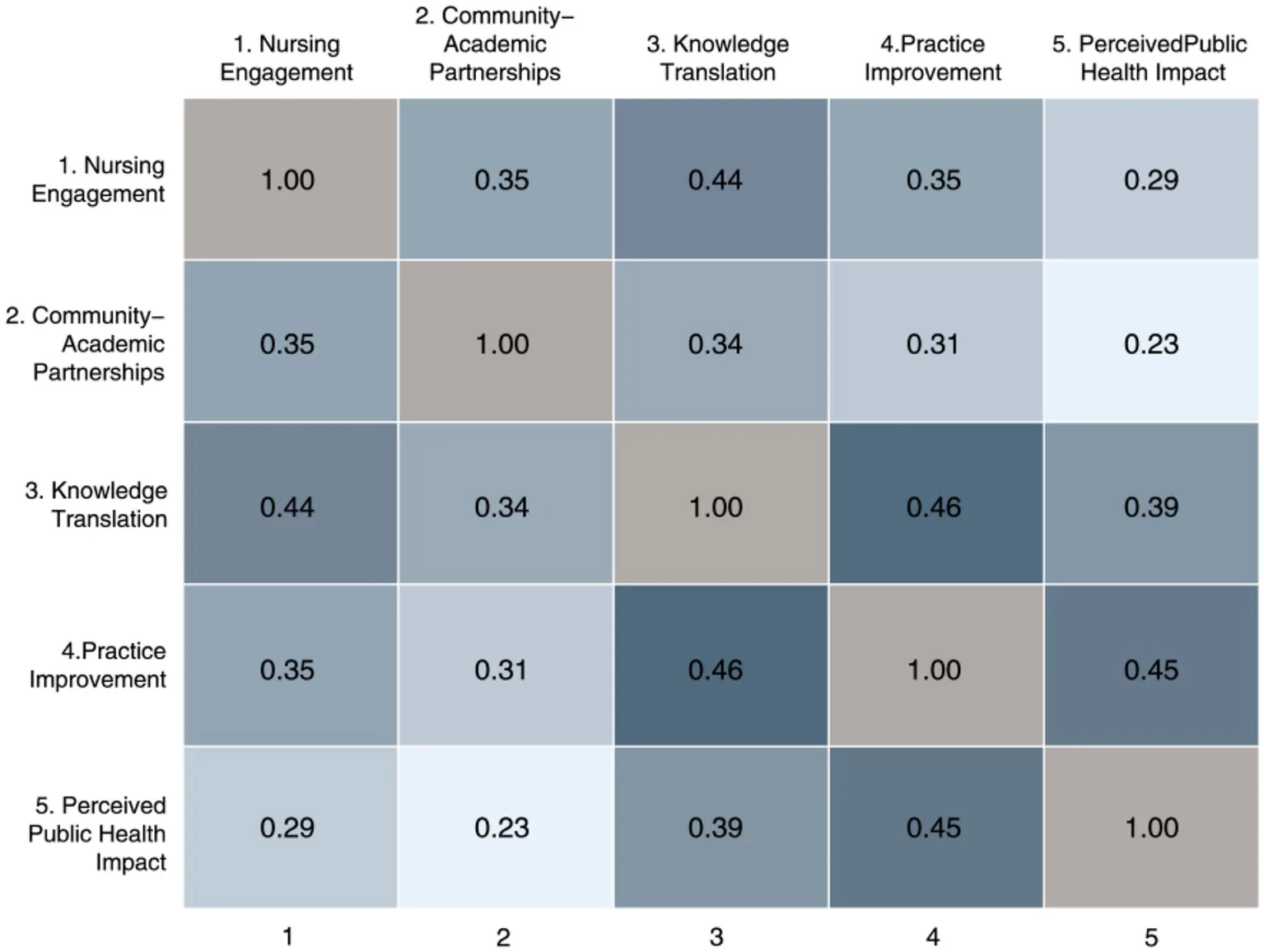
Correlation heatmap among the main constructs.

Pearson correlation analysis showed positive associations among all five constructs. Nurses’ work engagement was positively correlated with Knowledge Translation (*r* = 0.437, *p* < 0.001), Practice Improvement (*r* = 0.349, *p* < 0.001), Perceived Public Health Impact (*r* = 0.285, *p* < 0.001), and Community–Academic Partnerships (*r* = 0.347, *p* < 0.001). Community–Academic Partnerships was positively correlated with Knowledge Translation (*r* = 0.344, *p* < 0.001), Practice Improvement (*r* = 0.305, *p* < 0.001), and Perceived Public Health Impact (*r* = 0.230, *p* < 0.001). Knowledge Translation showed a moderate positive correlation with Practice Improvement (*r* = 0.457, *p* < 0.001) and Perceived Public Health Impact (*r* = 0.392, *p* < 0.001). Practice Improvement was also positively correlated with Perceived Public Health Impact (*r* = 0.449, *p* < 0.001). The complete correlation matrix is shown in Table 5. To provide a more direct visual summary of the association pattern, the correlation structure is presented as a heatmap in Figure 5.

**Table 5:** Pearson correlation matrix among the main constructs.

Construct	NE	CAP	KT	PI	PHI
Nurses’ work engagement	1.000				
Community–academic partnerships	0.347***	1.000			
Knowledge translation	0.437***	0.344***	1.000		
Practice improvement	0.349***	0.305***	0.457***	1.000	
Perceived public health impact	0.285***	0.230***	0.392***	0.449***	1.000

NE, Nurses’ work engagement; CAP, Community–academic partnerships; KT, knowledge translation; PI, practice improvement; PHI, perceived public health impact. Values are Pearson correlation coefficients.

***p < 0.001.

The heatmap visualizes Pearson correlations among Nurses’ work engagement, Community–Academic Partnerships, Knowledge Translation, Practice Improvement, and Perceived Public Health Impact. All correlations were positive and statistically significant, with stronger associations observed between Knowledge Translation and Practice Improvement and between Practice Improvement and Perceived Public Health Impact.

### 3.5 Adjusted regression results

Hierarchical regression analyses were conducted as supplementary covariate-adjusted estimates before testing the full latent-variable pathway model. In the model predicting Knowledge Translation, Nurses’ work engagement remained positively associated with Knowledge Translation after adjustment for demographic and professional covariates (*β* = 0.339, *p* < 0.001). Community–Academic Partnerships also remained positively associated with Knowledge Translation (*β* = 0.204, *p* < 0.001). The adjusted model explained 26.8% of the variance in Knowledge Translation.

In the model predicting Practice Improvement, Knowledge Translation was positively associated with Practice Improvement after adjustment (*β* = 0.373, p < 0.001), and Community–Academic Partnerships also showed a positive adjusted association (*β* = 0.156, *p* < 0.001). The adjusted model explained 26.1% of the variance in Practice Improvement.

In the model predicting Perceived Public Health Impact, Practice Improvement remained positively associated with Perceived Public Health Impact after adjustment (*β* = 0.417, *p* < 0.001). The adjusted model explained 24.9% of the variance in Perceived Public Health Impact. These adjusted regression results supported the direction of the proposed pathway and provided supplementary evidence before structural model estimation.

**Figure 6:**
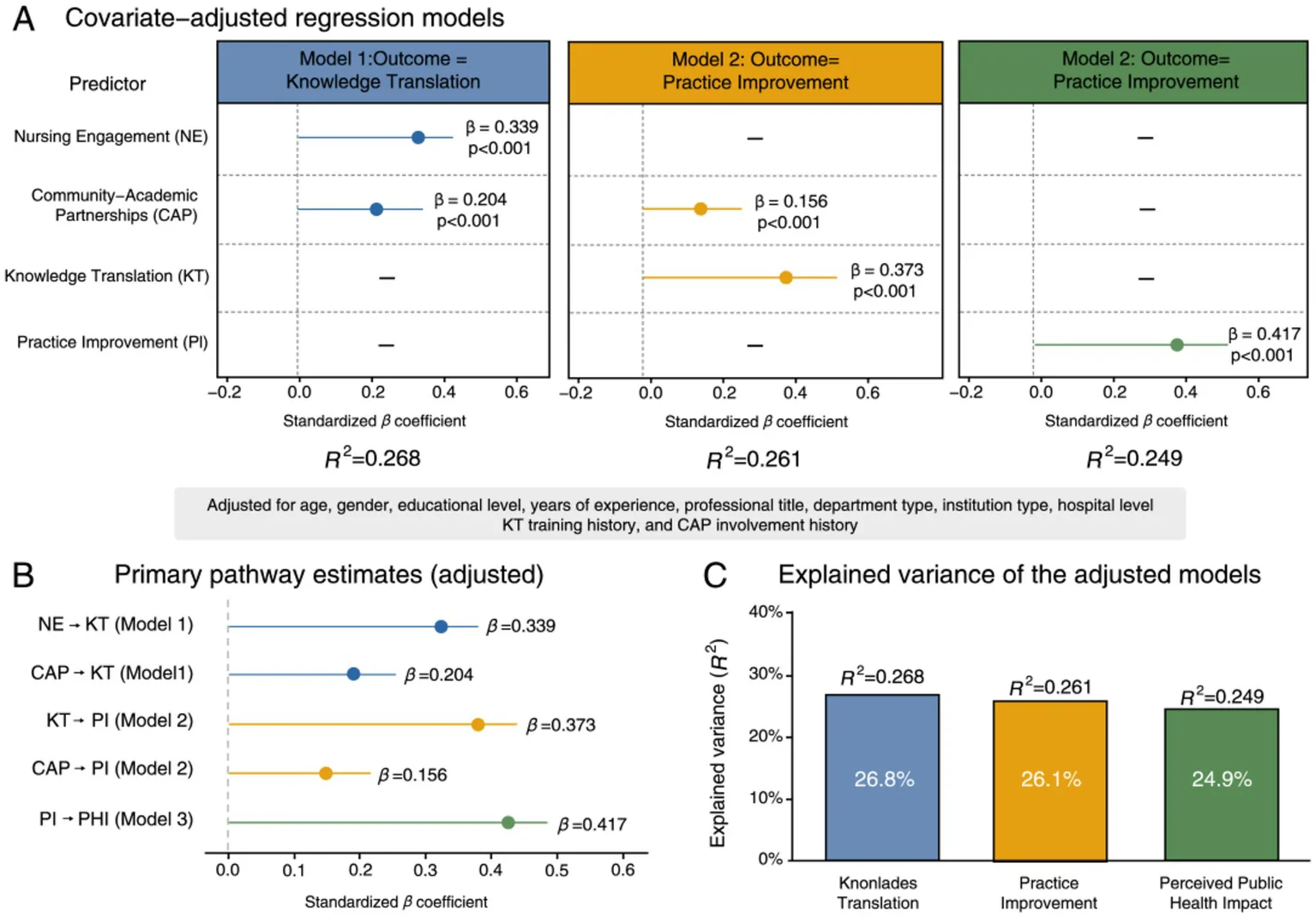
Covariate-adjusted regression estimates supporting the pre-specified pathway. **(A)** Covariate-adjusted regression models for Knowledge Translation, Practice Improvement, and Perceived Public Health Impact. **(B)** Primary adjusted pathway estimates. **(C)** Explained variance of the adjusted regression models.

Figure 6 presents the covariate-adjusted regression estimates supporting the pre-specified pathway before latent-variable structural model testing. In Model 1, Nurses’ work engagement and Community–Academic Partnerships were both positively associated with Knowledge Translation, explaining 26.8% of its variance. In Model 2, Knowledge Translation showed the strongest adjusted association with Practice Improvement, while Community–Academic Partnerships also remained positively associated with Practice Improvement; this model explained 26.1% of the variance. In Model 3, Practice Improvement was positively associated with Perceived Public Health Impact and explained 24.9% of its variance. These adjusted results were consistent with the proposed sequence from engagement and partnership exposure to knowledge translation, practice improvement, and perceived public health contribution.

### 3.6 Structural model results

**Figure 7:**
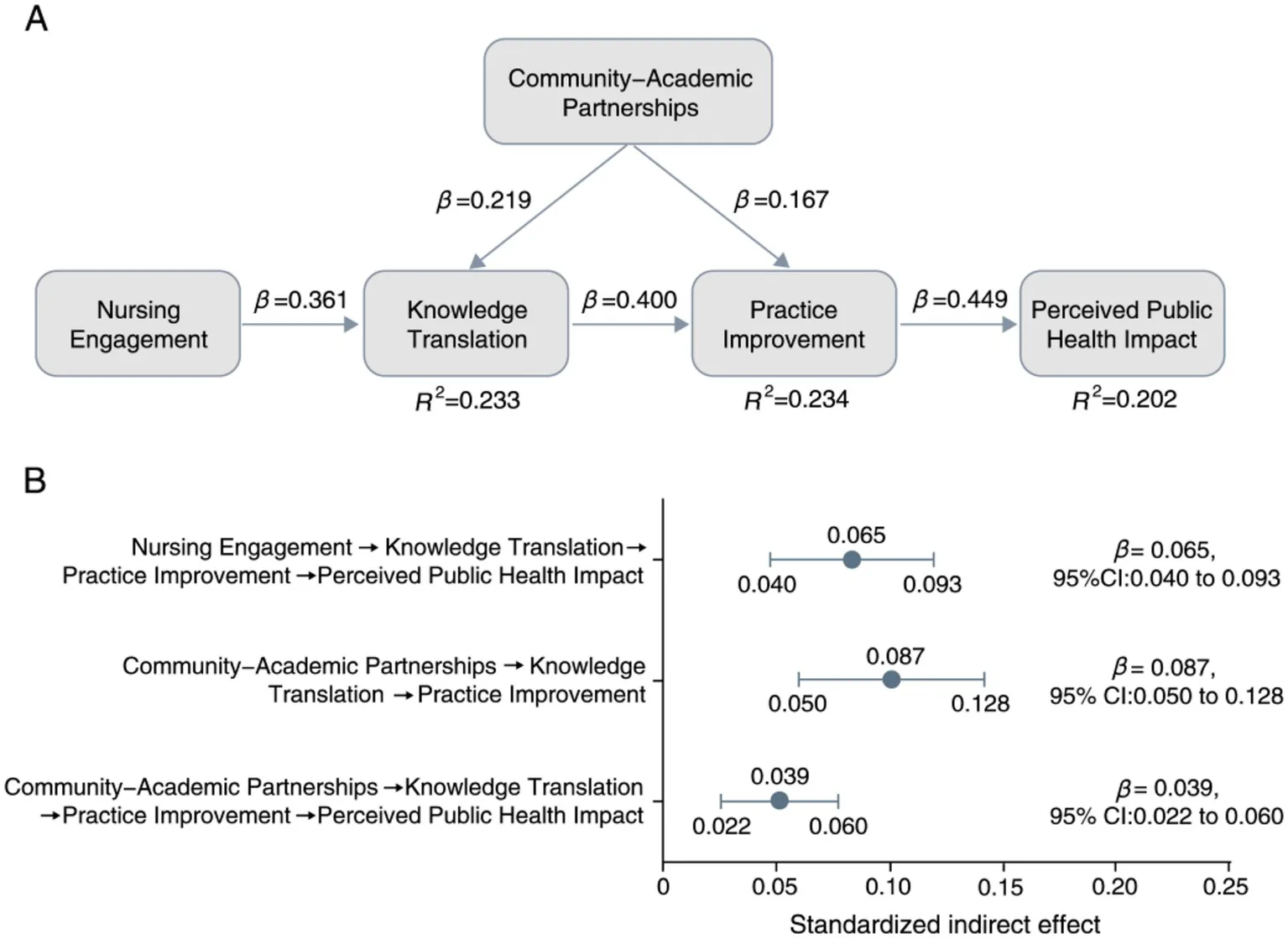
PLS-SEM structural pathway and indirect effects. **(A)** Estimated PLS-SEM structural pathway model. Standardized path coefficients are shown on the arrows, and *R*^2^ values are shown for endogenous constructs. **(B)** Bootstrapped indirect-effect estimates with 95% confidence intervals. The primary indirect pathway from nurses’ work engagement to perceived public health impact through knowledge translation and practice improvement was significant. Secondary indirect pathways involving community–academic partnerships were also supported.

PLS-SEM model and bootstrapped indirect effects are presented in Figure 7. Panel A shows the standardized structural paths and explained variance for endogenous constructs, while Panel B displays the indirect-effect estimates with 95% confidence intervals. The estimates describe a sequential pattern of associations linking work engagement and partnership exposure with knowledge translation, practice improvement, and perceived public health impact.

All pre-specified direct paths in the PLS-SEM model were positive and statistically significant. Nurses’ Work Engagement was positively associated with Knowledge Translation (*β* = 0.361, 95% CI: 0.267 to 0.448, *p* < 0.001). Community–Academic Partnerships was associated with Knowledge Translation (*β* = 0.219, 95% CI: 0.131 to 0.307, *p* < 0.001) and Practice Improvement (*β* = 0.167, 95% CI: 0.072 to 0.264, *p* < 0.001). Knowledge Translation was associated with Practice Improvement (*β* = 0.400, 95% CI: 0.313 to 0.478, *p* < 0.001), and Practice Improvement was associated with Perceived Public Health Impact (*β* = 0.449, 95% CI: 0.374 to 0.520, *p* < 0.001).

The structural model explained 23.3% of the variance in Knowledge Translation, 23.4% of the variance in Practice Improvement, and 20.2% of the variance in Perceived Public Health Impact. Collinearity was not a concern, as variance inflation factor values for the main structural predictors ranged from 1.00 to 1.14, below the pre-specified threshold of 3.30. The structural model and indirect-effect estimates are summarized in Table 6.

**Table 6:** Structural model and indirect-effect results.

Pathway/parameter	*β*	95% CI	*p*-value	VIF/*R*^2^
Direct structural paths				
Nurses’ work engagement → knowledge translation	0.361	0.267 to 0.448	<0.001	VIF = 1.14
Community–academic partnerships → knowledge translation	0.219	0.131 to 0.307	<0.001	VIF = 1.14
Knowledge translation → practice improvement	0.400	0.313 to 0.478	<0.001	VIF = 1.13
Community–academic partnerships → practice improvement	0.167	0.072 to 0.264	<0.001	VIF = 1.13
Practice improvement → perceived public health impact	0.449	0.374 to 0.520	<0.001	VIF = 1.00
Explained variance				
Knowledge translation	—	—	—	*R*^2^ = 0.233
Practice improvement	—	—	—	*R*^2^ = 0.234
Perceived public health impact	—	—	—	*R*^2^ = 0.202
Indirect effects				
Nurses’ work engagement → knowledge translation → practice improvement → perceived public health impact	0.065	0.040 to 0.093	<0.001	—
Community–academic partnerships → knowledge translation → practice improvement	0.087	0.050 to 0.128	<0.001	—
Community–academic partnerships → knowledge translation → practice improvement → perceived public health impact	0.039	0.022 to 0.060	<0.001	—

*β* values are standardized path coefficients or standardized indirect-effect estimates. CI, confidence interval; VIF, variance inflation factor. Indirect effects were estimated using nonparametric bootstrapping with 5,000 resamples. An indirect effect was considered statistically significant when the 95% bootstrap confidence interval did not include zero. *R*^2^ values represent the proportion of explained variance in endogenous constructs.

### 3.7 Indirect effects

The pre-specified primary indirect pathway was supported. Nurses’ Work Engagement had a positive indirect association with Perceived Public Health Impact through Knowledge Translation and Practice Improvement (*β* = 0.065, 95% CI: 0.040 to 0.093).

Secondary indirect pathways involving Community–Academic Partnerships were also supported. Community–Academic Partnerships was indirectly associated with Practice Improvement through Knowledge Translation (*β* = 0.087, 95% CI: 0.050 to 0.128), while the direct Community–Academic Partnerships → Practice Improvement path remained significant. Community–Academic Partnerships was also indirectly associated with Perceived Public Health Impact through Knowledge Translation and Practice Improvement (*β* = 0.039, 95% CI: 0.022 to 0.060). Because no direct partnership-to-impact path was estimated, this latter result is described as a significant indirect pathway rather than “full mediation”. Table 6 reports the indirect-effect estimates.

### 3.8 Sensitivity analyses

The sensitivity analyses supported the robustness of the main findings. In the subgroup of nurses with previous knowledge translation training, the main structural paths remained positive: Nurses’ work engagement → Knowledge Translation (*β* = 0.371), Community–Academic Partnerships → Knowledge Translation (*β* = 0.254), Knowledge Translation → Practice Improvement (*β* = 0.371), Community–Academic Partnerships → Practice Improvement (*β* = 0.157), and Practice Improvement → Perceived Public Health Impact (*β* = 0.415). In the subgroup without previous knowledge translation training, the same paths also remained positive, with coefficients of 0.332, 0.166, 0.428, 0.188, and 0.513, respectively. The adjusted regression models that included hospital level, department type, and nursing experience showed the same direction of association as the main model. Excluding the fastest 5% of valid responses according to the screening log did not materially change the direction or interpretation of the main structural paths.

**Figure 8:**
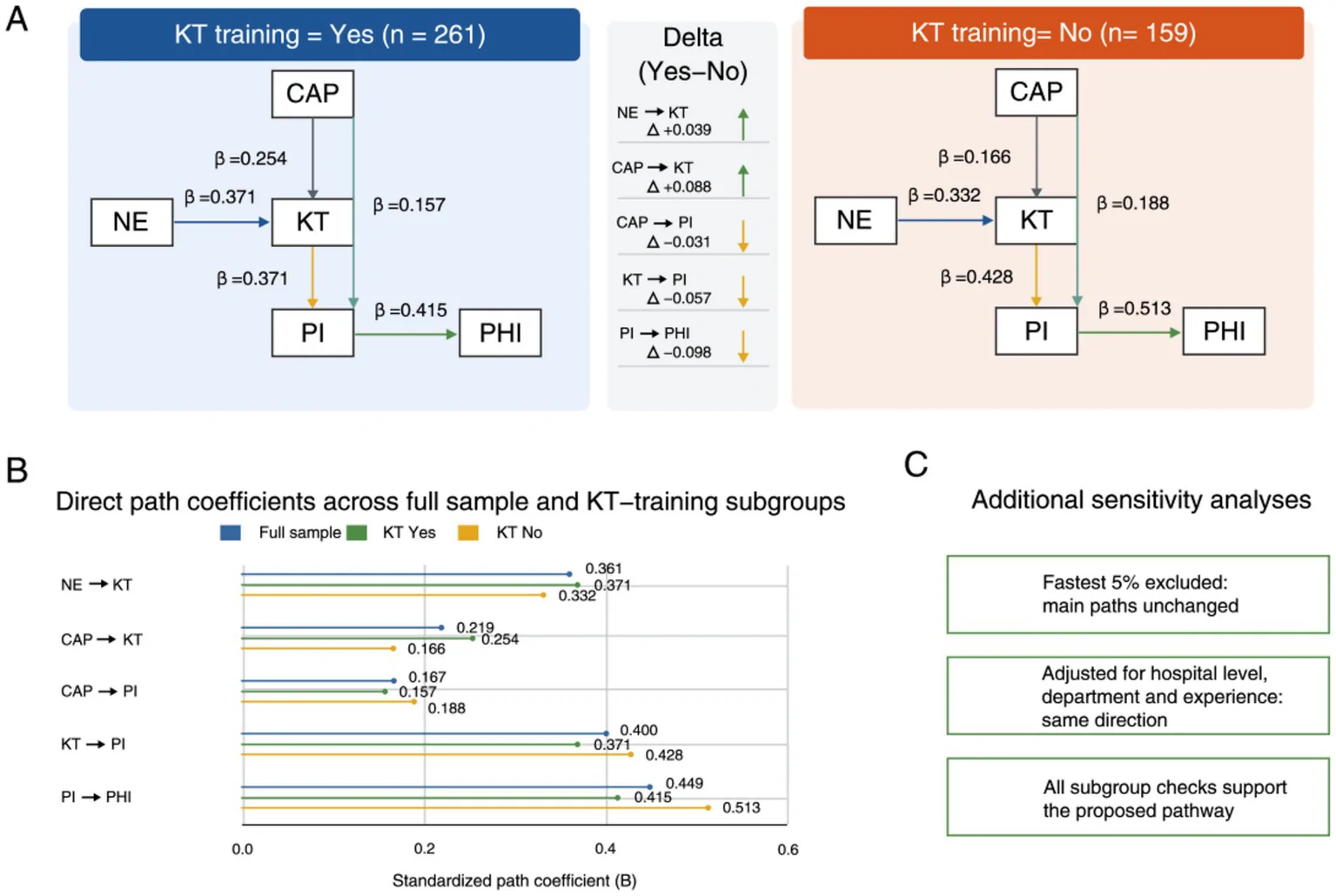
Robustness of structural pathways across knowledge-translation training subgroups. **(A)** Subgroup structural path coefficients according to previous knowledge-translation training. **(B)** Direct path coefficients across the full sample and KT-training subgroups. **(C)** Summary of additional sensitivity analyses.

Figure 8 summarizes the robustness of the main structural pathways across knowledge-translation training subgroups and additional sensitivity analyses. In both the KT-training subgroup and the non-training subgroup, all pre-specified structural paths remained positive, indicating directional consistency with the full-sample model. Nurses with previous KT training showed relatively stronger coefficients for Nurses’ work engagement → Knowledge Translation and Community–Academic Partnerships → Knowledge Translation, whereas the subgroup without previous KT training showed stronger downstream coefficients for Knowledge Translation → Practice Improvement and Practice Improvement → Perceived Public Health Impact. Additional sensitivity checks, including exclusion of the fastest 5% of valid responses and further adjustment for hospital level, department type, and years of nursing experience, did not materially alter the direction or interpretation of the main findings.

## 4 Discussion

This study examined associations among nurses’ work engagement, community–academic partnerships, knowledge translation, practice improvement, and nurses’ perceived public health impact in the post-pandemic era. The positive association between work engagement and knowledge translation is consistent with the possibility that engaged nurses are more likely to seek and apply professional evidence, although the cross-sectional data cannot determine directionality. Prior research has also linked work engagement with evidence-based practice implementation when organizational barriers are manageable (7). The present findings extend this literature by locating work engagement within a broader association-based pathway that includes knowledge translation and practice improvement.

Community–academic partnerships were positively associated with both knowledge translation and practice improvement. Partnership structures may provide access to academic resources, joint training, and shared improvement projects, particularly when collaboration is bidirectional and linked to practical problems (26, 27). In this study, partnership exposure was associated with the processes through which evidence was discussed, adapted, and applied. Knowledge translation was, in turn, associated with practice improvement in workflow, patient education, interdisciplinary communication, and follow-up. This interpretation is consistent with the Knowledge-to-Action perspective, which treats knowledge translation as an iterative process rather than simple information dissemination (28). These associations should be tested longitudinally before claims about organizational mechanisms are made.

Practice Improvement was positively associated with Perceived Public Health Impact. The outcome measured nurses’ perceived contribution to health awareness, preventive care, support for vulnerable populations, continuity of care, and responsiveness to public health needs; it was not an objective population-health indicator. The indirect-effect results were consistent with a sequential statistical pathway from work engagement to perceived impact through knowledge translation and practice improvement, and with corresponding pathways from partnership exposure. These patterns suggest that engaged staff and academic partnerships may be more useful when accompanied by structures that support evidence adaptation and practice change. Longitudinal and multi-source studies are required to evaluate temporal ordering and objective outcomes.

The post-pandemic context gives these findings additional relevance. Nurses have continued to carry responsibilities that extend beyond bedside care, including health education, prevention, follow-up support, continuity of care, and community-oriented response. Public health nursing has been increasingly discussed as a workforce and system priority after COVID-19 because health systems require stronger links between clinical care, community health needs, and preparedness capacity (29). In this context, nurses’ work engagement, knowledge translation, and practice improvement are not isolated professional concepts. They represent a practical route through which nursing teams may strengthen public health-oriented practice within hospitals, community health centers, and academic-practice collaborative settings. Several implications can be drawn from the findings. First, nurse managers should treat engagement as more than a morale indicator. Engagement should be connected to structured opportunities for evidence discussion, quality improvement, and implementation participation. Second, community–academic partnerships should be designed around concrete practice outputs, such as joint training, shared improvement projects, evidence adaptation workshops, and feedback loops between frontline nurses and academic partners. Third, knowledge translation should be operationalized as a routine practice process, not only as occasional continuing education. This is especially important because nurses in primary care and community-facing roles often manage rapidly changing public health tasks while maintaining continuity of routine care (30).

The findings also have methodological value. By combining measurement model assessment, adjusted regression, PLS-SEM, indirect-effect testing, and sensitivity analyses, this study offered a structured way to examine how individual engagement and organizational partnership conditions may work through knowledge translation and practice improvement. The calculated R^2^ values indicate that the structural model explained a meaningful but non-exhaustive portion of variance across the endogenous constructs. Crucially, the proposed pathway remains inherently susceptible to unmeasured occupational and organizational factors. Variables such as staffing adequacy, leadership support, job control, professional burnout, and overarching organizational climate were not included in the current analytical framework. Because these omitted structural variables can simultaneously influence multiple constructs within our model, they represent potent sources of residual confounding. Consequently, the adjusted associations and pathway coefficients must be interpreted with appropriate caution. Future analytical designs must explicitly integrate these contextual confounders to partial out their overlapping effects.

This study has several limitations. First, the cross-sectional design precludes causal inference and cannot confirm the temporal ordering of the proposed pathway; reciprocal or alternative models remain plausible. Second, all constructs were self-reported in a single survey, and common method bias cannot be excluded. Third, Perceived Public Health Impact measured perceived contribution rather than population-level outcomes. Fourth, purposive convenience sampling through established networks may have introduced selection and compliance biases and limits generalizability. Several additional limitations should be acknowledged. Participants were clustered within 12 institutions, but clustering was not modeled; therefore, standard errors may have been underestimated. Furthermore, the measurement and structural models were evaluated in the same sample, and independent external validation is warranted before generalization.

Despite these limitations, the study provides a coherent empirical account of how nurses’ work engagement may be converted into public health-oriented practice value through knowledge translation and practice improvement. The findings suggest that post-pandemic nursing development should not rely only on individual professional commitment. It also requires partnership structures, implementation capacity, and practice systems that allow evidence to move into daily nursing work. This conclusion is consistent with evidence-based nursing scholarship, which emphasizes that better outcomes depend not only on available evidence but also on the organizational and professional processes that enable nurses to use it (31).

## 5 Conclusion

In this cross-sectional sample, nurses’ work engagement, community–academic partnerships, knowledge translation, and practice improvement formed a coherent pattern of associations with nurses’ perceived public health impact. Work engagement and partnership exposure were indirectly associated with perceived impact through knowledge translation and practice improvement. These findings suggest that workforce engagement may be most useful when organizations also provide access to evidence, academic–practice collaboration, and structured opportunities for practice improvement. Because the data were cross-sectional, self-reported, and drawn through purposive convenience sampling, the proposed sequence should be interpreted as a statistical pathway rather than a causal mechanism. Longitudinal, multisite studies using objective outcomes and site-level analyses are needed to test the direction and generalizability of these associations.

## Data Availability

The raw data supporting the conclusions of this article will be made available by the authors, without undue reservation.
