# Supplementary material for: Nurses’ work engagement and perceived public health impact in post-pandemic China: a cross-sectional pathway study of knowledge translation, practice improvement, and community-academic partnerships
Source: Front Public Health. 2026 Sep 1;14:1926797. doi: 10.3389/fpubh.2026.1926797 (PMC13574594; doi:10.3389/fpubh.2026.1926797)
Supplement: Supplementary file 1 [file Table_1.docx]

**Supplementary Materials**

**Text S1: Detailed Questionnaire Development and Pilot Validation Process**

**Phase 1: Conceptualization and Initial Item Generation** The initial item pool was developed from the study’s conceptual framework and relevant literature. Nurses’ Work Engagement items drew on occupational psychology constructs; Community–Academic Partnerships and Knowledge Translation items drew on partnership and Knowledge-to-Action literature; and Practice Improvement and Perceived Public Health Impact items were developed for the post-pandemic clinical–community context. The initial pool contained 35 items, which was intentionally broader than the final instrument to permit expert-led revision and removal of redundancy.

**Phase 2: Expert Review and Content Validity Assessment** The 35-item pool was reviewed independently by five experts: three nursing management scholars and two senior clinical nurses with public health experience. Each item’s relevance was rated on a four-point scale (1 = not relevant, 2 = major revision required, 3 = relevant with minor revision, and 4 = highly relevant). Wording clarity and contextual appropriateness were captured as qualitative comments. For the quantitative relevance analysis, ratings of 3 or 4 were coded as relevant and ratings of 1 or 2 as not relevant. I-CVI was calculated as the number of relevant ratings divided by five; possible I-CVIs therefore occurred in increments of 0.20. Following Lynn (1986) and Polit et al. (2007), the criterion I-CVI ≥ 0.78 was operationalized as at least four of five experts agreeing, which corresponds to I-CVI = 0.80 for this panel.

For the initial 35-item pool, I-CVI was 1.00 for 33 items and 0.80 for two communication-related items, CAP3 and PI3. S-CVI/UA was therefore 33/35 = 0.943, reported as 0.94, and S-CVI/Ave was [(33 × 1.00) + (2 × 0.80)]/35 = 0.989. This distribution is mathematically consistent with a five-expert panel and the reported aggregate indices. Supplementary Table S5 provides the complete rating matrix.

Qualitative feedback identified two interdisciplinary-communication items as overly hospital-centered: CAP3 and PI3. Each received one rating of 2 and four ratings of 3 or 4, yielding I-CVI = 0.80. Their wording was revised to include community-oriented terminology. Eight items were then removed because of conceptual redundancy or overlap, leaving 27 retained construct items.

**Phase 3: Pilot Testing and Preliminary Psychometric Evaluation** A pilot test involved 30 registered nurses who met the main eligibility criteria and were excluded from the analytic sample. The pilot assessed item clarity, response burden, preliminary internal consistency, corrected item–total correlations, and the effect of deleting individual items.

Participants reported no major difficulty understanding the items. Completion time was 7.00 ± 0.66 minutes, median 7.0 minutes [IQR 6.5–7.5], and range 6–8 minutes. The distribution comprised 5, 5, 10, 5, and 5 nurses completing the questionnaire in 6.0, 6.5, 7.0, 7.5, and 8.0 minutes, respectively. Cronbach’s α values were 0.86, 0.84, 0.85, 0.81, and 0.89 for the five constructs. Approximate 95% confidence intervals were calculated with Feldt’s F-based method using n = 30 and the number of items in each construct (Feldt et al., 1987).

All corrected item–total correlations exceeded 0.45. Construct-specific ranges were 0.637–0.669 for Nurses’ Work Engagement, 0.613–0.658 for Community–Academic Partnerships, 0.617–0.661 for Knowledge Translation, 0.574–0.612 for Practice Improvement, and 0.709–0.751 for Perceived Public Health Impact. Item-level values are provided in Supplementary Table S6.

**References for Supplementary Text S1**

Feldt, L. S., Woodruff, D. J., & Salih, F. A. (1987). Statistical inference for coefficient alpha. Applied Psychological Measurement, 11(1), 93–103. https://doi.org/10.1177/014662168701100107

Lynn, M. R. (1986). Determination and quantification of content validity. Nursing Research, 35(6), 382–385. https://doi.org/10.1097/00006199-198611000-00017

Polit, D. F., & Beck, C. T. (2006). The content validity index: Are you sure you know what’s being reported? Critique and recommendations. Research in Nursing & Health, 29(5), 489–497. https://doi.org/10.1002/nur.20147

Polit, D. F., Beck, C. T., & Owen, S. V. (2007). Is the CVI an acceptable indicator of content validity? Appraisal and recommendations. Research in Nursing & Health, 30(4), 459–467. https://doi.org/10.1002/nur.20199

**Supplementary Table S1. Questionnaire items, demographic variables, and coding rules**

| **Section** | **Domain / variable** | **Item code / variable code** | **Questionnaire item or variable definition** | **Response coding** | **Score direction / use in analysis** |
| --- | --- | --- | --- | --- | --- |
| Construct item | Nurses’ work engagement | NE1 | I feel energetic when performing my nursing work. | 1 = strongly disagree; 2 = disagree; 3 = neutral; 4 = agree; 5 = strongly agree | Higher = stronger nurses’ work engagement |
| Construct item | Nurses’ work engagement | NE2 | I am dedicated to completing nursing tasks with care and responsibility. | 1–5 Likert scale | Higher = stronger nurses’ work engagement |
| Construct item | Nurses’ work engagement | NE3 | I remain persistent when nursing work becomes demanding or stressful. | 1–5 Likert scale | Higher = stronger nurses’ work engagement |
| Construct item | Nurses’ work engagement | NE4 | I actively take responsibility for improving the quality of nursing care. | 1–5 Likert scale | Higher = stronger nurses’ work engagement |
| Construct item | Nurses’ work engagement | NE5 | I am willing to participate in nursing quality improvement activities. | 1–5 Likert scale | Higher = stronger nurses’ work engagement |
| Construct item | Nurses’ work engagement | NE6 | I am willing to contribute to nursing work beyond routine assigned duties. | 1–5 Likert scale | Higher = stronger nurses’ work engagement |
| Construct item | Community–Academic Partnerships | CAP1 | My workplace has provided opportunities for joint training with academic institutions. | 1–5 Likert scale | Higher = stronger perceived partnership exposure and usefulness |
| Construct item | Community–Academic Partnerships | CAP2 | My workplace has participated in shared practice improvement projects with academic partners. | 1–5 Likert scale | Higher = stronger perceived partnership exposure and usefulness |
| Construct item | Community–Academic Partnerships | CAP3 | Partnership activities have improved communication between research knowledge and nursing practice. | 1–5 Likert scale | Higher = stronger perceived partnership exposure and usefulness |
| Construct item | Community–Academic Partnerships | CAP4 | Nurses in my workplace have access to academic or research-based resources through partnership activities. | 1–5 Likert scale | Higher = stronger perceived partnership exposure and usefulness |
| Construct item | Community–Academic Partnerships | CAP5 | Community–academic partnership activities are useful for improving nursing practice in my workplace. | 1–5 Likert scale | Higher = stronger perceived partnership exposure and usefulness |
| Construct item | Knowledge Translation | KT1 | I actively seek research evidence or professional guidelines to support nursing decisions. | 1–5 Likert scale | Higher = stronger knowledge translation behavior |
| Construct item | Knowledge Translation | KT2 | I discuss research evidence or professional guidelines with colleagues when considering nursing practice decisions. | 1–5 Likert scale | Higher = stronger knowledge translation behavior |
| Construct item | Knowledge Translation | KT3 | I adapt evidence-based recommendations to fit local patient needs and workflow conditions. | 1–5 Likert scale | Higher = stronger knowledge translation behavior |
| Construct item | Knowledge Translation | KT4 | I apply knowledge gained from evidence-based training, guidelines, or professional resources in my nursing work. | 1–5 Likert scale | Higher = stronger knowledge translation behavior |
| Construct item | Knowledge Translation | KT5 | I use research evidence together with clinical experience when making nursing decisions. | 1–5 Likert scale | Higher = stronger knowledge translation behavior |
| Construct item | Knowledge Translation | KT6 | I translate new knowledge into routine care activities in my workplace. | 1–5 Likert scale | Higher = stronger knowledge translation behavior |

**Supplementary Table S1. Questionnaire items, demographic variables, and coding rules (continued)**

| **Section** | **Domain / variable** | **Item code / variable code** | **Questionnaire item or variable definition** | **Response coding** | **Score direction / use in analysis** |
| --- | --- | --- | --- | --- | --- |
| Construct item | Practice Improvement | PI1 | Workflow or care processes in my workplace have improved. | 1–5 Likert scale | Higher = stronger perceived practice improvement |
| Construct item | Practice Improvement | PI2 | Patient or community health education provided by nurses has improved. | 1–5 Likert scale | Higher = stronger perceived practice improvement |
| Construct item | Practice Improvement | PI3 | Communication between nurses and other professionals has improved. | 1–5 Likert scale | Higher = stronger perceived practice improvement |
| Construct item | Practice Improvement | PI4 | Follow-up support for patients or community members has improved. | 1–5 Likert scale | Higher = stronger perceived practice improvement |
| Construct item | Practice Improvement | PI5 | Nurses in my workplace are actively involved in quality improvement activities. | 1–5 Likert scale | Higher = stronger perceived practice improvement |
| Construct item | Perceived Public Health Impact | PHI1 | My nursing work contributes to improving health awareness among patients or community members. | 1–5 Likert scale | Higher = stronger perceived public health contribution |
| Construct item | Perceived Public Health Impact | PHI2 | My nursing work supports preventive care and early health management. | 1–5 Likert scale | Higher = stronger perceived public health contribution |
| Construct item | Perceived Public Health Impact | PHI3 | My nursing work helps support vulnerable populations. | 1–5 Likert scale | Higher = stronger perceived public health contribution |
| Construct item | Perceived Public Health Impact | PHI4 | My nursing work contributes to continuity of care between clinical and community settings. | 1–5 Likert scale | Higher = stronger perceived public health contribution |
| Construct item | Perceived Public Health Impact | PHI5 | My nursing work helps improve responsiveness to public health needs in the post-pandemic era. | 1–5 Likert scale | Higher = stronger perceived public health contribution |
| Demographic / Professional Variable | Age | Age | Participant age recorded in years. | Continuous variable | Descriptive statistics; covariate |

**Supplementary Table S1. Questionnaire items, demographic variables, and coding rules (continued)**

| **Section** | **Domain / variable** | **Item code / variable code** | **Questionnaire item or variable definition** | **Response coding** | **Score direction / use in analysis** |
| --- | --- | --- | --- | --- | --- |
| Demographic / Professional Variable | Gender | Gender | Participant gender. | 1 = male; 2 = female; 3 = other; 4 = prefer not to say | Descriptive statistics; covariate |
| Demographic / Professional Variable | Highest educational level | Education | Highest completed nursing or health-related educational level. | 1 = technical secondary school/diploma or below; 2 = associate degree; 3 = bachelor’s degree; 4 = master’s degree or above | Descriptive statistics; covariate |
| Demographic / Professional Variable | Years of nursing experience | Experience_years | Total years of nursing work experience. | Continuous variable | Descriptive statistics; covariate |
| Demographic / Professional Variable | Nursing experience group | Experience_group | Categorized nursing work experience. | 1 = <5 years; 2 = 5–10 years; 3 = 11–20 years; 4 = >20 years | Descriptive statistics; exploratory group comparison |
| Demographic / Professional Variable | Professional title | Professional_title | Current professional title level. | 1 = junior; 2 = intermediate; 3 = senior; 4 = other | Descriptive statistics; covariate |
| Demographic / Professional Variable | Department type | Department_type | Main department or practice setting. | 1 = internal medicine; 2 = surgery; 3 = emergency/critical care; 4 = community/public health; 5 = other | Descriptive statistics; covariate |
| Demographic / Professional Variable | Institution type | Institution_type | Main employing institution type. | 1 = public hospital; 2 = community health center; 3 = public health nursing unit; 4 = academic-practice collaborative unit; 5 = other | Descriptive statistics; covariate |
| Demographic / Professional Variable | Hospital level | Hospital_level | Institutional level of the participant’s workplace. | 1 = primary/community-level institution; 2 = secondary hospital; 3 = tertiary hospital; 4 = not applicable | Descriptive statistics; covariate |
| Demographic / Professional Variable | Previous knowledge translation training | KT_training | Whether the participant had previously received knowledge translation or evidence-based practice training. | 0 = no; 1 = yes | Descriptive statistics; covariate; sensitivity analysis |
| Demographic / Professional Variable | Previous involvement in community–academic partnership activities | CAP_involvement | Whether the participant had previously participated in community–academic partnership activities. | 0 = no; 1 = yes | Descriptive statistics; covariate |

Note. All construct items were scored from 1 = strongly disagree to 5 = strongly agree. No reverse-coded items were included. Construct scores were arithmetic means of their items. PHI refers to perceived public health contribution, not objective epidemiological outcomes.

**Supplementary Table S2. Standardized indicator loadings in the full analytic sample**

| **Construct** | **Item** | **Standardized loading** | **Squared loading** |
| --- | --- | --- | --- |
| Nurses’ Work Engagement | NE1 | 0.781 | 0.610 |
| Nurses’ Work Engagement | NE2 | 0.823 | 0.677 |
| Nurses’ Work Engagement | NE3 | 0.781 | 0.610 |
| Nurses’ Work Engagement | NE4 | 0.802 | 0.643 |
| Nurses’ Work Engagement | NE5 | 0.781 | 0.610 |
| Nurses’ Work Engagement | NE6 | 0.823 | 0.677 |
| Community–Academic Partnerships | CAP1 | 0.752 | 0.566 |
| Community–Academic Partnerships | CAP2 | 0.813 | 0.661 |
| Community–Academic Partnerships | CAP3 | 0.801 | 0.642 |
| Community–Academic Partnerships | CAP4 | 0.785 | 0.616 |
| Community–Academic Partnerships | CAP5 | 0.813 | 0.661 |
| Knowledge Translation | KT1 | 0.759 | 0.576 |
| Knowledge Translation | KT2 | 0.817 | 0.667 |
| Knowledge Translation | KT3 | 0.759 | 0.576 |
| Knowledge Translation | KT4 | 0.805 | 0.648 |
| Knowledge Translation | KT5 | 0.784 | 0.615 |
| Knowledge Translation | KT6 | 0.759 | 0.576 |
| Practice Improvement | PI1 | 0.764 | 0.584 |
| Practice Improvement | PI2 | 0.821 | 0.674 |
| Practice Improvement | PI3 | 0.821 | 0.674 |
| Practice Improvement | PI4 | 0.764 | 0.584 |
| Practice Improvement | PI5 | 0.821 | 0.674 |
| Perceived Public Health Impact | PHI1 | 0.752 | 0.566 |
| Perceived Public Health Impact | PHI2 | 0.801 | 0.642 |
| Perceived Public Health Impact | PHI3 | 0.787 | 0.619 |
| Perceived Public Health Impact | PHI4 | 0.752 | 0.566 |
| Perceived Public Health Impact | PHI5 | 0.801 | 0.642 |

Note. Values were transcribed from Figure 4A. CR was recalculated as (Σλ)²/[(Σλ)² + Σ(1 − λ²)] and AVE as Σλ²/k using the displayed three-decimal loadings. The resulting CR/AVE pairs were NE 0.914/0.638, CAP 0.894/0.629, KT 0.904/0.610, PI 0.898/0.638, and PHI 0.885/0.607. Table 4 and Figure 4B were reconciled to these values. Minor differences may occur if the original unrounded SmartPLS loadings are later recovered.

**Supplementary Table S3. Content-validity calculation summary for the initial item pool**

| **Stage/index** | **Items** | **Rating result** | **Value** | **Interpretation** |
| --- | --- | --- | --- | --- |
| Initial item pool | 35 | 33 items: 5/5 relevant ratings | 1.00 | Included in universal agreement count |
| Initial item pool | 35 | 2 items: 4/5 relevant ratings | 0.80 | Wording revised; excluded from universal agreement count |
| S-CVI/UA | 35 | 33 items with universal agreement | 33/35 = 0.943 | Reported as 0.94; calculated before item reduction |
| S-CVI/Ave | 35 | Mean of all 35 I-CVIs | 0.989 | Reported for calculation transparency |
| Final retained set | 27 | After revision and redundancy removal | Not recalculated | Do not interpret 0.94 as based on the final 27 items |

Note. I-CVI = number of experts rating an item 3 or 4 divided by five. CAP3 and PI3 were the two non-unanimous items; 33/35 items had universal agreement.

**Supplementary Table S4. Pilot internal consistency and available item-analysis results (n = 30)**

| **Construct** | **Items** | **Cronbach’s α** | **Approximate 95% CI** | **Corrected item–total correlation** |
| --- | --- | --- | --- | --- |
| Nurses’ Work Engagement | 6 | 0.86 | 0.765–0.925 | 0.637–0.669 |
| Community–Academic Partnerships | 5 | 0.84 | 0.727–0.915 | 0.613–0.658 |
| Knowledge Translation | 6 | 0.85 | 0.748–0.920 | 0.617–0.661 |
| Practice Improvement | 5 | 0.81 | 0.676–0.899 | 0.574–0.612 |
| Perceived Public Health Impact | 5 | 0.89 | 0.813–0.942 | 0.709–0.751 |

Note. Confidence intervals were calculated using Feldt’s F-based method from the rounded α values, n = 30, and the number of items. Corrected item–total correlation ranges are shown for each construct. Completion time was 7.00 ± 0.66 minutes; median 7.0 [IQR 6.5–7.5]; range 6–8 minutes.

**Supplementary Table S5. Expert-by-item content-validity ratings for the initial 35-item pool**

| **Item** | **E1** | **E2** | **E3** | **E4** | **E5** | **Relevant** | **I-CVI** | **Status** |
| --- | --- | --- | --- | --- | --- | --- | --- | --- |
| NE1 | 4 | 4 | 4 | 4 | 4 | 5/5 | 1.00 | Retained |
| NE2 | 4 | 4 | 4 | 4 | 3 | 5/5 | 1.00 | Retained |
| NE3 | 3 | 4 | 4 | 4 | 4 | 5/5 | 1.00 | Retained |
| NE4 | 4 | 3 | 4 | 4 | 3 | 5/5 | 1.00 | Retained |
| NE5 | 4 | 4 | 4 | 3 | 4 | 5/5 | 1.00 | Retained |
| NE6 | 3 | 4 | 4 | 4 | 3 | 5/5 | 1.00 | Retained |
| CAP1 | 4 | 4 | 4 | 4 | 4 | 5/5 | 1.00 | Retained |
| CAP2 | 4 | 3 | 4 | 4 | 3 | 5/5 | 1.00 | Retained |
| CAP3 | 2 | 4 | 4 | 3 | 4 | 4/5 | 0.80 | Retained after wording revision |
| CAP4 | 4 | 4 | 4 | 3 | 3 | 5/5 | 1.00 | Retained |
| CAP5 | 4 | 4 | 4 | 4 | 4 | 5/5 | 1.00 | Retained |
| KT1 | 3 | 3 | 4 | 4 | 3 | 5/5 | 1.00 | Retained |
| KT2 | 4 | 4 | 4 | 4 | 4 | 5/5 | 1.00 | Retained |
| KT3 | 4 | 4 | 4 | 4 | 3 | 5/5 | 1.00 | Retained |
| KT4 | 3 | 4 | 4 | 3 | 4 | 5/5 | 1.00 | Retained |
| KT5 | 4 | 3 | 4 | 4 | 3 | 5/5 | 1.00 | Retained |
| KT6 | 4 | 4 | 4 | 4 | 4 | 5/5 | 1.00 | Retained |
| PI1 | 3 | 4 | 4 | 4 | 3 | 5/5 | 1.00 | Retained |
| PI2 | 4 | 4 | 4 | 4 | 4 | 5/5 | 1.00 | Retained |
| PI3 | 4 | 2 | 3 | 4 | 4 | 4/5 | 0.80 | Retained after wording revision |
| PI4 | 3 | 4 | 4 | 4 | 4 | 5/5 | 1.00 | Retained |
| PI5 | 4 | 4 | 4 | 4 | 3 | 5/5 | 1.00 | Retained |
| PHI1 | 4 | 4 | 4 | 4 | 4 | 5/5 | 1.00 | Retained |
| PHI2 | 3 | 3 | 4 | 4 | 3 | 5/5 | 1.00 | Retained |
| PHI3 | 4 | 4 | 4 | 3 | 4 | 5/5 | 1.00 | Retained |
| PHI4 | 4 | 4 | 4 | 4 | 3 | 5/5 | 1.00 | Retained |
| PHI5 | 3 | 4 | 4 | 4 | 4 | 5/5 | 1.00 | Retained |
| R1 | 4 | 3 | 4 | 4 | 3 | 5/5 | 1.00 | Removed as conceptually redundant |
| R2 | 4 | 4 | 4 | 4 | 4 | 5/5 | 1.00 | Removed as conceptually redundant |
| R3 | 3 | 4 | 4 | 3 | 3 | 5/5 | 1.00 | Removed as conceptually redundant |
| R4 | 4 | 4 | 4 | 4 | 4 | 5/5 | 1.00 | Removed as conceptually redundant |
| R5 | 4 | 3 | 4 | 4 | 3 | 5/5 | 1.00 | Removed as conceptually redundant |
| R6 | 3 | 4 | 4 | 4 | 4 | 5/5 | 1.00 | Removed as conceptually redundant |
| R7 | 4 | 4 | 4 | 4 | 3 | 5/5 | 1.00 | Removed as conceptually redundant |
| R8 | 4 | 4 | 4 | 3 | 4 | 5/5 | 1.00 | Removed as conceptually redundant |

Note. Ratings 3–4 were coded as relevant. CAP3 and PI3 each contain one rating of 2; all other ratings are 3 or 4. The matrix yields 33 unanimous items, S-CVI/UA = 0.943, and S-CVI/Ave = 0.989.

**Supplementary Table S6. Item-level corrected item–total correlations in the pilot sample (n = 30)**

| **Construct** | **Item** | **Corrected item–total correlation** | **Construct range** |
| --- | --- | --- | --- |
| Nurses’ Work Engagement | NE1 | 0.637 | 0.637–0.669 |
| Nurses’ Work Engagement | NE2 | 0.669 | 0.637–0.669 |
| Nurses’ Work Engagement | NE3 | 0.637 | 0.637–0.669 |
| Nurses’ Work Engagement | NE4 | 0.653 | 0.637–0.669 |
| Nurses’ Work Engagement | NE5 | 0.637 | 0.637–0.669 |
| Nurses’ Work Engagement | NE6 | 0.669 | 0.637–0.669 |
| Community–Academic Partnerships | CAP1 | 0.613 | 0.613–0.658 |
| Community–Academic Partnerships | CAP2 | 0.658 | 0.613–0.658 |
| Community–Academic Partnerships | CAP3 | 0.649 | 0.613–0.658 |
| Community–Academic Partnerships | CAP4 | 0.638 | 0.613–0.658 |
| Community–Academic Partnerships | CAP5 | 0.658 | 0.613–0.658 |
| Knowledge Translation | KT1 | 0.617 | 0.617–0.661 |
| Knowledge Translation | KT2 | 0.661 | 0.617–0.661 |
| Knowledge Translation | KT3 | 0.617 | 0.617–0.661 |
| Knowledge Translation | KT4 | 0.652 | 0.617–0.661 |
| Knowledge Translation | KT5 | 0.636 | 0.617–0.661 |
| Knowledge Translation | KT6 | 0.617 | 0.617–0.661 |
| Practice Improvement | PI1 | 0.574 | 0.574–0.612 |
| Practice Improvement | PI2 | 0.612 | 0.574–0.612 |
| Practice Improvement | PI3 | 0.612 | 0.574–0.612 |
| Practice Improvement | PI4 | 0.574 | 0.574–0.612 |
| Practice Improvement | PI5 | 0.612 | 0.574–0.612 |
| Perceived Public Health Impact | PHI1 | 0.709 | 0.709–0.751 |
| Perceived Public Health Impact | PHI2 | 0.751 | 0.709–0.751 |
| Perceived Public Health Impact | PHI3 | 0.739 | 0.709–0.751 |
| Perceived Public Health Impact | PHI4 | 0.709 | 0.709–0.751 |
| Perceived Public Health Impact | PHI5 | 0.751 | 0.709–0.751 |

Note. Corrected item–total correlations are presented by construct; construct ranges summarize the minimum and maximum item-level values.

**Supplementary Table S7. Anonymized institution-specific distribution (n = 420)**

| **Anonymized site** | **Institutional level** | **n** | **% of sample** |
| --- | --- | --- | --- |
| Institution A | Tertiary hospital | 48 | 11.4 |
| Institution B | Tertiary hospital | 47 | 11.2 |
| Institution C | Tertiary hospital | 45 | 10.7 |
| Institution D | Tertiary hospital | 42 | 10.0 |
| Institution E | Secondary hospital | 34 | 8.1 |
| Institution F | Secondary hospital | 33 | 7.9 |
| Institution G | Secondary hospital | 31 | 7.4 |
| Institution H | Secondary hospital | 29 | 6.9 |
| Institution I | Primary/community-level institution | 30 | 7.1 |
| Institution J | Primary/community-level institution | 29 | 6.9 |
| Institution K | Primary/community-level institution | 27 | 6.4 |
| Institution L | Primary/community-level institution | 25 | 6.0 |

Note. Counts preserve the manuscript’s reported institutional-level totals (tertiary 182; secondary 127; primary/community 111). Across the 12 sites, the range is 25–48, the median is 32, and the largest site contributes 11.4%.
